# A bioactive molecule made by unusual salvage of radical SAM enzyme byproduct 5-deoxyadenosine blurs the boundary of primary and secondary metabolism

**DOI:** 10.1016/j.jbc.2021.100621

**Published:** 2021-03-31

**Authors:** Johanna Rapp, Pascal Rath, Joachim Kilian, Klaus Brilisauer, Stephanie Grond, Karl Forchhammer

**Affiliations:** 1 Interfaculty Institute of Microbiology and Infection Medicine, Microbiology/Organismic Interactions, Eberhard Karls Universität Tübingen, Tübingen, Germany; 2 Institute of Organic Chemistry, Eberhard Karls Universität Tübingen, Tübingen, Germany; 3 Center for Plant Molecular Biology, Eberhard Karls Universität Tübingen, Tübingen, Germany

**Keywords:** 5-deoxyadenosine salvage, 5-deoxyribose, 7-deoxysedoheptulose biosynthesis, enzyme promiscuity, *S-*adenosylmethionine, radical SAM enzymes, cyanobacteria, secondary metabolism, carbohydrate biosynthesis, GC–MS, 5dAdo, 5-deoxyadenosine, 5dAdo^•^, 5-deoxyadenosylradical, 5dR, 5-deoxyribose, 5dR-1P, 5-deoxyribose 1-phosphate, 5dRu-1P, 5-deoxyribulose 1-phosphate, 7dSh, 7-deoxysedoheptulose, ATCC, American Type Culture Collection, DHAP, dihydroxyacetone phosphate, HAD, haloacid dehalogenase, MSP, methionine salvage pathway, MTA, 5-methylthioadenosine, MtnN, MTA nucleosidase, MtnP, MTA phosphorylase, MTR, methylthioribose, MTR-1P, methylthioribose-1-phosphate, SAH, *S*-adenosylhomocysteine, SAM, *S*-adenosyl-l-methionine

## Abstract

5-Deoxyadenosine (5dAdo) is the byproduct of many radical *S*-adenosyl-l-methionine enzyme reactions in all domains of life. 5dAdo is also an inhibitor of the radical *S*-adenosyl-l-methionine enzymes themselves, making it necessary for cells to construct pathways to recycle or dispose of this toxic metabolite. However, the specific pathways involved have long remained unexplored. Recent research demonstrated a growth advantage in certain organisms by using 5dAdo or intermediates as a sole carbon source and elucidated the corresponding salvage pathway. We now provide evidence using supernatant analysis by GC–MS for another 5dAdo recycling route. Specifically, in the unicellular cyanobacterium *Synechococcus elongatus* PCC 7942 (*S. elongatus*), the activity of promiscuous enzymes leads to the synthesis and excretion first of 5-deoxyribose and subsequently of 7-deoxysedoheptulose. 7-Deoxysedoheptulose is an unusual deoxy-sugar, which acts as an antimetabolite of the shikimate pathway, thereby exhibiting antimicrobial and herbicidal activity. This strategy enables organisms with small genomes and lacking canonical gene clusters for the synthesis of secondary metabolites, like *S. elongatus*, to produce antimicrobial compounds from primary metabolism and enzymatic promiscuity. Our findings challenge the view of bioactive molecules as sole products of secondary metabolite gene clusters and expand the range of compounds that microorganisms can deploy to compete for their ecological niche.

*S*-Adenosyl-l-methionine (SAM; AdoMet), which is formed by ATP and the amino acid methionine, is an essential cofactor of various enzymatic reactions in all domains of life. SAM can serve as a methyl group donor for the methylation of DNA, RNA, and proteins in reactions that release *S*-adenosylhomocysteine (SAH) as a byproduct (1). SAM can also serve as an aminopropyl donor for polyamine synthesis and as a homoserine lactone donor for the synthesis of quorum-sensing compound *N*-acetylhomoserine lactone, both of which result in the release of 5-methylthioadenosine (MTA). Furthermore, SAM is a source of the 5-deoxyadenosylradical (5dAdo^•^), which is formed by the activity of radical SAM enzymes (1, 2, 3, 4, 5). 5dAdo^•^ is formed by the reductive cleavage of SAM and can abstract a hydrogen atom from its substrate to form a substrate radical as well as 5-deoxyadenosine (5dAdo), which is released as a byproduct (3, 6). Radical SAM enzymes, a superfamily with over 100,000 members, are present in all domains of life (2, 7). They are catalyzing various complex chemical reactions, including sulfur insertion, anaerobic oxidations, unusual methylations, and ring formations (8). Prominent members are, for example, involved in biotin, thiamine, and lipoate biosynthesis. Other members are involved in DNA repair or in the biosynthesis of secondary metabolites, for example, antibiotics (3). MTA, SAH, and 5dAdo are product inhibitors of these reactions (8, 9, 10, 11, 12). Therefore, and because of the high bioenergetic costs of these compounds, salvage pathways are necessary. SAH is rescued *via* the methionine cycle (13). MTA salvage *via* the methionine salvage pathway (MSP) is also well characterized (14, 15) (Fig. 1*B*). In the classical and aerobic MSP, MTA is either processed by a two-step reaction by the MTA nucleosidase (MtnN), followed by a phosphorylation by the methylthioribose (MTR) kinase or by the MTA phosphorylase (MtnP). The subsequent reactions consist of a dehydration (MtnB, methylthioribose-1-phosphate [MTR-1P] dehydratase), enolization and phosphorylation (either by MtnC: DK-MTP-1P enolase/phosphatase or by MtnW: DK-MTP-1P enolase and MtnX: HK-MTPene-1P phosphatase; DK-MTP-1P: 2,3-diketo-5-methylthiopentyl-1-phosphate, HK-MTPene-1P: 2-hydroxy-3-keto-5-methylthiopentenyl-1-phosphate), deoxygenation (MtnD: acireductone dioxygenase), and a final transamination step (MtnE: aminotransferase).

**Figure 1:**
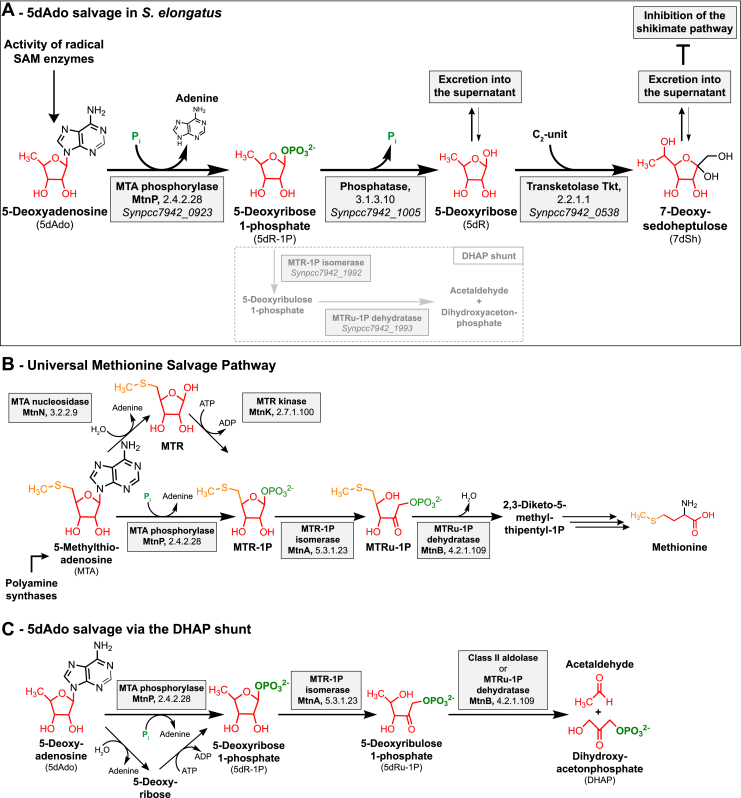
**Overview of the 5dAdo and MTA salvage pathways.***A,* 5dAdo salvage in *Synechococcus elongatus via* the excretion of the bioactive deoxy-sugars 5dR and 7dSh (this study). 5dR-1P is partially also metabolized *via* the DHAP shunt (shown by *dashed line*), especially under low carbon conditions. *B,* universal methionine salvage pathway (16). *C,* 5dAdo salvage *via* the DHAP shunt (13, 19). MTA, 5-methylthioadenosine; MTR, methylthioribose; MTRu-1P, methylthioribulose-1P; SAM, *S*-adenosyl-l-methionine.

Despite the high abundance of radical SAM enzymes and thereby of 5dAdo, less is known about 5dAdo salvage. *In vitro* experiments showed that 5dAdo can be processed by a two-step reaction, in which 5dAdo is cleaved by promiscuous MtnN resulting in the release of adenine and 5-deoxyribose (5dR) (9, 10). The subsequent phosphorylation of 5dR by MTR kinase results in the formation of 5-deoxyribose 1-phosphate (5dR-1P). The second option is the direct conversion of 5dAdo into 5dR-1P and adenine *via* promiscuous MtnP (16). Therefore, it is suggested that 5dAdo salvage is paralogous to the MSP and is driven by the promiscuous activity of the enzymes of the MSP (17). Recently, a pathway for 5dR salvage was elucidated in *Bacillus thuringiensis* involving the sequential activity of a kinase, an isomerase, and a class II aldolase, which are encoded by a specific gene cluster (18). The authors propose that 5dR is phosphorylated to 5dR-1P, which is then isomerized into 5-deoxyribulose 1-phosphate (5dRu-1P) and subsequently cleaved by an aldolase into acetaldehyde and dihydroxyacetone phosphate (DHAP) for primary metabolism. In organisms that lack the specific gene cluster, the cleavage of 5dAdo into DHAP and acetaldehyde is proposed to occur *via* the promiscuous activity of enzymes of the MSP. In support of this hypothesis, it was shown that *Arabidopsis thaliana* DEP1, an MTR-1P dehydratase of the MSP, is promiscuous and can also cleave 5dRu-1P into DHAP and acetaldehyde, suggesting that a specific aldolase is not required for 5dAdo salvage (18). In agreement with this, the promiscuous activity of MSP enzymes in the 5dAdo salvage was recently reported in *Methanocaldococcus jannaschii* (*M. jannaschii*), where methylthioribose 1-phosphate isomerase uses MTR-1P, 5dR-1P, and 5dR as substrates (19). Only recently, 5dAdo was shown to be processed to DHAP and acetaldehyde by a gene cluster consisting of the first enzymes of the MSP as well as a class II aldolase in *Rhodospirillum rubrum* and pathogenic *Escherichia coli* strains, in a process termed “DHAP shunt” (13) (Fig. 1*C*).

In our previous work, we isolated the rare deoxy-sugar—namely, 7-deoxysedoheptulose (7-deoxy-d-*altro*-2-heptulose, 7-deoxy-sedoheptulose [7dSh])—from the supernatant of the unicellular cyanobacterium *Synechococcus elongatus* PCC 7942 (*S. elongatus*) (20). This compound showed bioactivity toward various prototrophic organisms, for example, other cyanobacteria, especially *Anabaena variabilis* American Type Culture Collection (ATCC) 29413 (*A. variabilis*), *Saccharomyces*, and *Arabidopsis*. It blocks the shikimate pathway presumably by inhibiting the enzyme dehydroquinate synthase (20). Because of the streamlined genome of *S. elongatus* and the lack of specific gene clusters for secondary metabolite synthesis (21, 22), the pathway for 7dSh formation remained enigmatic. Of note, 7dSh was also isolated from the supernatant of *Streptomyces setonensis* (20, 23), but the synthesis pathway remained unresolved. Therefore, we speculated that 7dSh synthesis might involve promiscuous enzymes of primary metabolism. Enzyme promiscuity, the ability of an enzyme to use various substrates, is especially important for organisms with a small genome. Previously, it was described that the marine cyanobacterium *Prochlorococcus* uses a single promiscuous enzyme that can transform up to 29 different ribosomally synthesized peptides into an arsenal of polycyclic bioactive products (24). As from the 7dSh-containing supernatant of *S. elongatus*, we in addition isolated the deoxy-sugar 5dR. We hypothesized that 5dR could serve as a precursor molecule of 7dSh (20). *In vitro*, 5dR can serve as a substrate for a transketolase-based reaction, in which a C_2_ unit is transferred to the C_5_ unit leading to the formation of 7dSh (20).

In this work, we identified the pathway for 7dSh biosynthesis, which involves a new salvage route for 5dAdo resulting in the release of 5dR and 7dSh into the culture medium (Fig. 1*A*). Therefore, *S. elongatus* can synthesize a bioactive compound from the products of the primary metabolism simply by using promiscuous enzymes.

## Results

### 5dR and 7dSh accumulation in supernatants of *S. elongatus* is strongly promoted by CO_2_ supplementation

**Figure 2:**
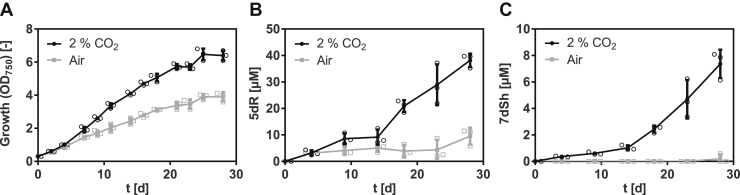
**5-Deoxyribose (5dR) and 7-deoxysedoheptulose (7dSh) accumulation in the supernatant of *Synechococcus elongatus* is strongly promoted by high CO**_**2**_**concentrations.***S. elongatus* cultures aerated either with ambient air (*gray squares*) or with air supplemented with 2% CO_2_ (*black dots*). *A,* over time, growth of *S. elongatus* (indicated by an absorbance at 750 nm). Over time, concentration of 5dR (*B*) or 7dSh (*C*) in the supernatant of *S. elongatus* cultures. Note the different values of the *y*-axis. Data shown represent mean and standard deviation of three independent biological replicates.

Previously, we estimated the content of 7dSh in the supernatant of *S. elongatus* cultures *via* a bioassay based on the size of the inhibition zone of *A. variabilis* exposed to the supernatant of *S. elongatus* (20). To quantify the amounts of 5dR and 7dSh formed by *S. elongatus*, we developed a GC–MS–based method that enables the detection and absolute quantification of low micromolar concentrations of these metabolites in the culture supernatant. In cultures supplemented with 2% CO_2_ (Fig. 2, *black dots*), 5dR gradually accumulated during growth (Fig. 2, *A* and *B*), whereas 7dSh accumulation only occurred during a later growth phase (Fig. 2*C*). After 30 days of cultivation, the supernatant contained four times more 5dR than 7dSh. Under ambient air conditions (Fig. 2, *gray squares*), small amounts of 5dR were formed, whereas 7dSh could not be detected (Fig. 2, *B* and *C*), despite that the optical density of the air-supplied cultures in the final stage of the experiment reached values similar to those of the CO_2_-supplemented cultures, where 7dSh accumulation could be detected (Fig. 2*A*). This suggests that the formation of the deoxy-sugars is not only growth phase dependent but also related to a specific metabolic state.

**Figure 3:**
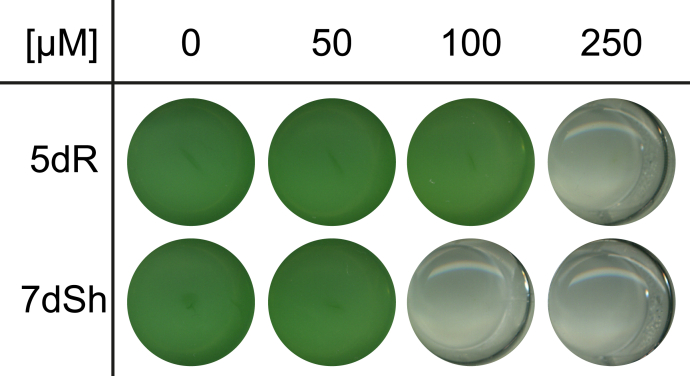
**5-Deoxyribose (5dR) and 7-deoxysedoheptulose (7dSh) are inhibiting the growth of the producer strain.** Effect of different concentrations of 5dR and 7dSh on the growth of *Synechococcus elongatus*. The cultures were inoculated at an optical density of 750 nm of 0.1 in 1 ml BG11 medium in the absence (0) or the presence of either 5dR or 7dSh at the indicated concentrations and grown in a 24-well plate for 3 days. The experiment was performed in triplicates. The results of one replicate are shown.

To gain further insights into 5dR/7dSh metabolism, we measured the intracellular concentration of 5dR and 7dSh over the whole cultivation process. Surprisingly, only small intracellular amounts, close to detection limit of either deoxy-sugar, could be detected (Fig. S1), whereas the extracellular concentration gradually increased. This strongly suggests that extracellular 5dR/7dSh accumulation is not because of cell lysis but involves immediate secretion after their formation. Removal of these metabolites from the cytoplasm is probably essential for *S. elongatus* as both molecules showed growth inhibition toward the producer strain at elevated concentrations (Fig. 3). 7dSh is bactericidal at concentrations of 100 μM, whereas 5dR is bacteriostatic at concentrations of 250 μM.

### 5dR is a precursor molecule for 7dSh biosynthesis *in vivo*

**Figure 4:**
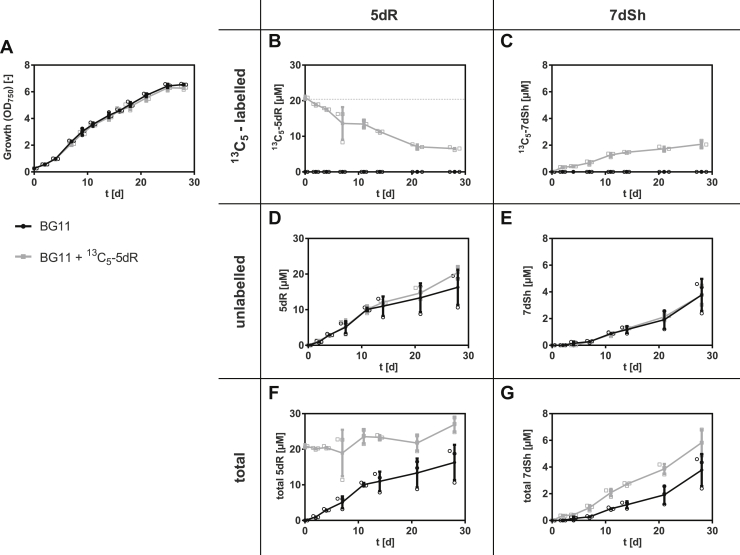
**5-Deoxyribose (5dR) is the precursor molecule of 7-deoxysedoheptulose (7dSh).** Effects of ^13^C_5_-5dR supplementation over the time on the growth of *Synechococcus elongatus* (*A*) or on the concentration of ^13^C_5_-5dR (*B*), ^13^C_5_-7dSh (*C*), unlabeled 5dR (*D*), unlabeled 7dSh (*E*), total 5dR (*F*), and total 7dSh (*G*) in the culture supernatant. About 20 μM ^13^C_5_-5dR (indicated by *dashed line*) was added at the beginning of the cultivation (*gray squares*). Control cultures (*black dots*) were cultivated in BG11 without supplemented ^13^C_5_-5dR. All cultures were aerated with air supplemented with 2% CO_2_. Values shown in the graphs represent mean and standard deviation of three biological replicates.

In our previous work, we reported the *in vitro* synthesis of 7dSh by converting 5dR into 7dSh by a transketolase-based reaction with hydroxypyruvate as a C_2_ unit donor (20). To determine whether 5dR might also be a precursor molecule for 7dSh *in vivo*, a 5dR-feeding experiment was performed (Fig. 4). To unambiguously distinguish the naturally formed and the supplemented 5dR, uniformly labeled [U-^13^C_5_]-5dR (^13^C_5_-5dR) was synthesized and added at a final concentration of 20 μM to *S. elongatus* cultures at the beginning of the cultivation. The concentration of labeled (Fig. 4, *B* and *C*), unlabeled, (Fig. 4, *D* and *E*), and the total amount of 5dR and 7dSh (Fig. 4, *F* and *G*) was determined by GC–MS at different time points over a period of 30 days.

Neither the growth of *S. elongatus* nor the excretion of unlabeled and intracellular synthesized 5dR and 7dSh was affected by the addition of exogenous ^13^C_5_-5dR (Fig. 4, *A*, *D*, and E). We found that ^13^C_5_-5dR is taken up by the cultures as its concentration in the supernatant continuously decreased (Fig. 4*B*, *gray squares*). Already within 2 days, ^13^C_5_-7dSh could be detected in the supernatant of these cultures (Fig. 4*C*, *gray squares*), clearly proving that ^13^C_5_-7dSh was formed from the precursor molecule ^13^C_5_-5dR. However, only a small amount of exogenously added ^13^C_5_-5dR was converted into 7dSh. At the end of the experiment, 10% of the initially applied ^13^C_5_-5dR (20 μM) was converted into ^13^C_5_-7dSh (∼2 μM). Around 30% of ^13^C_5_-5dR remained in the supernatant (6.5 μM). The residual amount is assumed to be metabolized *via* (an)other pathway(s). Because unlabeled 5dR was excreted at the same time as ^13^C_5_-5dR was taken up (Fig. 4, *B* and *D*), 5dR must be imported and exported in parallel.

### 5dAdo as a precursor molecule of 7dSh

**Figure 5:**
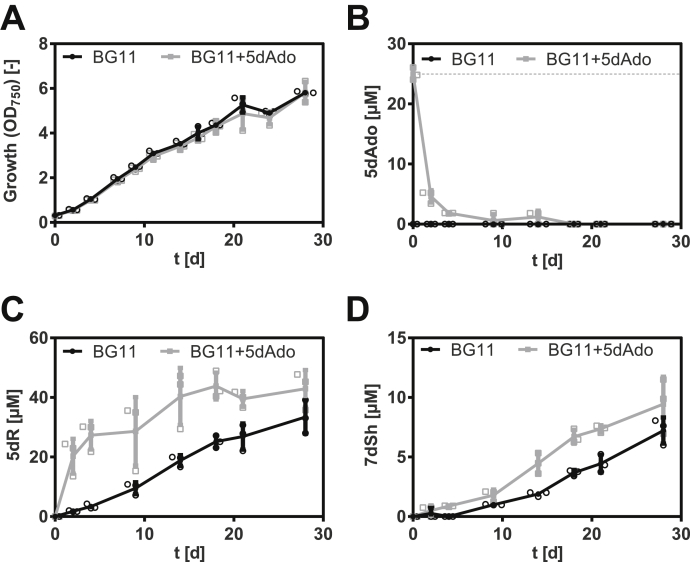
**5-Deoxyadenosine (5dAdo) feeding experiment.** Effect of 5dAdo supplementation on the growth of *Synechococcus elongatus* (*A*) or on the concentration of 5dAdo (*B*), 5-deoxyribose (5dR) (*C*), and 7-deoxy-sedoheptulose (7dSh) (*D*) in the culture supernatant. About 25 μM 5dAdo (indicated by *dashed line*) was added at the beginning of the cultivation (*gray squares*). Control cultures (*black dots*) were cultivated in BG11 in the absence of exogenous 5dAdo. All cultures were aerated with air supplemented with 2% CO_2_. Note the different values of the *y*-axis. Values shown in the graphs represent mean and standard deviation of three biological replicates.

Next, we asked the question where 5dR is derived from. This drew our attention to 5dAdo, a byproduct of radical SAM enzymes (3). The compound has to be removed because of its intracellular toxicity (9), and its cleavage can result in the formation of 5dR (9, 18) (Fig. 1*C*). To prove that 7dSh is formed from 5dAdo salvage in *S. elongatus*, 5dAdo-feeding experiments were performed, and the supernatants were analyzed by GC–MS (Fig. 5). Notably, the growth of *S. elongatus* was not affected by supplementation with 5dAdo, which was taken up very quickly (Fig. 5, *A* and *B*). After 4 days, almost all 5dAdo was taken up. A control experiment showed that the rapid decline in the amount of 5dAdo in the supernatant was not caused by the instability of 5dAdo in the medium. Feeding of the cells with 5dAdo immediately led to an enhanced accumulation of 5dR in the culture supernatant (Fig. 5*C*). After 14 days, 7dSh levels in 5dAdo-supplemented cultures were clearly enhanced compared with control cultures (Fig. 5*D*), supporting our hypothesis that 5dAdo is a precursor molecule of 7dSh. However, only about half of the supplemented 5dAdo (initial concentration: 25 μM) was converted into 5dR and 7dSh: at the end of the experiment, the 5dR concentration in the supplemented cultures was increased by around 10 μM and that of 7dSh by 2 μM, suggesting additional pathway(s) for 5dAdo salvage.

**Figure 6:**
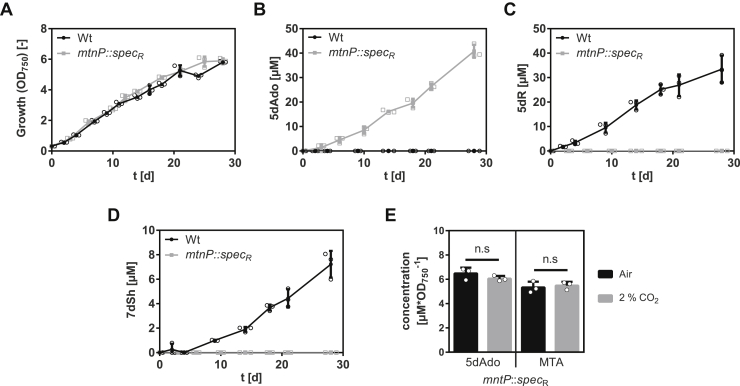
**5-Deoxyadenosine (5dAdo) is cleaved by 5-methylthioadenosine (MTA) phosphorylase (MtnP) and then metabolized into 5-deoxyribose (5dR) and 7-deoxysedoheptulose (7dSh) in *Synechococcus elongatus* at high CO**_**2**_**concentrations.** Growth (*A*), concentrations of 5dAdo (*B*), 5dR (*C*), and 7dSh (*D*) in the supernatant of *S. elongatus* wildtype (*black dots*) or *mtnP*::*spec*_*R*_ mutant (*gray squares*). All cultures were aerated with air supplemented with 2% CO_2_. Note the different values of the *y*-axis. *E,* 5dAdo and MTA concentrations in the supernatant of the *mtnP*::*spec*_*R*_ mutant normalized on the optical density after 11 days of cultivation (cultures were either aerated with atmospheric air [*black*] or with air supplemented with 2% CO_2_ [*gray*]). Significant differences between the concentrations of 5dAdo or MTA during cultivation at 2% CO_2_ and ambient air were analyzed by using an unpaired *t* test (∗*p* < 0.05; ∗∗*p* < 0.01; and ∗∗∗*p* < 0.001). Values shown in the graphs represent mean and standard deviation of three biological replicates. ns, not significant.

5dAdo is known to be cleaved by either the MtnN or the MtnP (9, 10, 16). The former reaction leads to the release of adenine and 5dR, whereas the latter leads to phosphate-dependent release of adenine and 5dR-1P. In *S. elongatus,* no homologous gene for a MtnN was found, but gene *Synpcc7942_0923* is annotated as a MtnP. Therefore, an insertion mutant was generated *via* the replacement of the gene by an antibiotic resistance cassette (*S. elongatus mtnP*::*spec*_*R*_). Polar effects because of the insertion of the antibiotic resistance cassette were excluded by monitoring the expression of the genes with a semiquantitative RT-PCR (Fig. S2). Under conditions favorable for 5dR/7dSh production, the mutant grew like the wildtype (Fig. 6*A*). A GC–MS analysis of the culture supernatant revealed that the mutant neither excreted 5dR nor 7dSh (Fig. 6, *C* and *D*). Instead, while undetectable in the supernatant of the wildtype strain, 5dAdo strongly accumulated in the supernatant of *S. elongatus mtnP*::*spec*_*R*_ cultures (Fig. 6*B*). This confirmed that 5dR/7dSh are derived from 5dAdo in a MtnP-dependent manner. Because of the detoxification *via* excretion, the *mtnP*::*spec*_*R*_ mutant escapes the toxic effect of 5dAdo and does not show any growth disadvantage (Fig. 6*A*). It has previously been reported that a *mtnP* knockout mutant in *Saccharomyces cerevisiae* as well as MtnP-deficient mammalian tumor cells excreted MTA (25, 26). Both MTA and 5dAdo are known to be cleaved by MtnP (16). Consistently, the *mtnP*::*spec*_*R*_ mutant excretes MTA as well as 5dAdo (Fig. 6*E*). Since 5dR/7dSh formation strongly depends on elevated CO_2_ conditions, we measured the amount of 5dAdo and MTA in cultures of the *mtnP*::*spec*_*R*_ mutant supplied with ambient air or with air enriched with 2% CO_2_. However, the amounts of excreted 5dAdo and MTA (normalized to the optical density of the cultures) were almost identical under both conditions (Fig. 6*E*). This clearly indicates that 5dAdo salvage *via* 5dR/7dSh formation and excretion at high CO_2_ conditions is not triggered by an increased synthesis of the precursor molecule 5dAdo compared with ambient CO_2_ concentrations. Rather, it appears that 5dAdo is actively metabolized into 5dR/7dSh under elevated CO_2_ conditions, whereas 5dAdo salvage under ambient CO_2_ conditions is conducted by (an)other pathway(s). Since the MTA formation is also unaltered (Fig. 6*E*), we conclude that 5dAdo salvage *via* 5dR/7dSh formation is not triggered by an enhanced demand of MTA salvage *via* the MSP pathway.

### 5dR and 7dSh formation is not ubiquitous

To clarify how widespread the synthesis of 7dSh or 5dR is in cyanobacteria, we analyzed the supernatants of other cyanobacterial strains *via* GC–MS (*Synechococcus* sp. PCC 6301, *Synechococcus* sp. PCC 7002, *Synechococcus* sp. PCC 6312, *Synechococcus* sp. PCC 7502, *Synechocystis* sp. PCC 6803, *A. variabilis* ATCC 29413, *Nostoc punctiforme* ATCC 29133, and *Anabaena* sp. PCC 7120). Only in three of five *Synechococcus* strains, the deoxy-sugars 5dR and 7dSh were detectable. All the other strains accumulated neither 5dR nor 7dSh. In the freshwater strain *Synechococcus* sp. PCC 6301, the amounts of 7dSh and 5dR were in a similar concentration range to those in *S. elongatus*. This is not surprising since the genome of *Synechococcus* sp. PCC 6301 is nearly identical to that of *S. elongatus* PCC 7942 (27). Very small amounts of 5dR and 7dSh were detected in the marine strain *Synechococcus* sp. PCC 7002. In *S. setonensis*, which was shown to produce 7dSh (20, 23), we detected 113 ± 7 μM 7dSh but no 5dR in the supernatant of cultures grown for 7 days.

### 5dAdo cleavage is strictly dependent on phosphorylase activity

**Figure 7:**
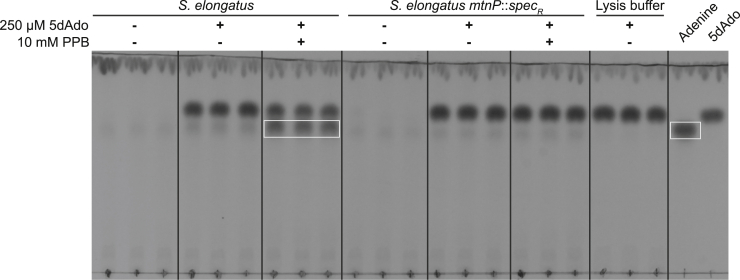
**5-Deoxyadenosine (5dAdo) cleavage in *Synechococcus elongatus* is phosphate dependent.** Crude extracts from *S. elongatus* or *S. elongatus mtnP*::s*pec*_*R*_ were incubated with 5dAdo in the presence or the absence of potassium phosphate buffer (PPB) and then analyzed *via* TLC on silica gel. 5dAdo (*R*_f_ = 0.68) and adenine (*R*_f_ = 0.76) analytes were visualized *via* absorption at 254 nm. Pure adenine and 5dAdo were used as standards (*right*). Spots corresponding to adenine are highlighted with a *white box*. Three independent replicates are shown for each condition. The stability of 5dAdo in the buffer is shown with the lysis buffer control.

To reveal whether 5dAdo is converted to 5dR *via* MtnP activity, crude extracts of *S. elongatus* wildtype and MtnP-deficient *mtnP*::*spec*_*R*_ mutant cells were incubated with 5dAdo in the presence or the absence of potassium phosphate buffer. Analysis of the extracts *via* TLC revealed that 5dAdo cleavage and, thereby, adenine release is strictly dependent on the presence of phosphate (Fig. 7, *white label*) and only occurred in wildtype cell extracts but not in extracts of *mtnP*::*spec*_*R*_ mutant cells. Therefore, 5dAdo cleavage in *S. elongatus* is strictly dependent on the presence of the MtnP. Other enzymes, for example, purine nucleosidase phosphorylases (28), apparently do not process 5dAdo in the cell extract. This result implies that the first product of 5dAdo cleavage is 5dR-1P, which is subsequently converted into 5dR. 5dR-1P seemed quite stable because LC-MS analysis revealed that a compound with an *m/z* ratio that corresponds to the sum formula of 5dR-1P ([M + H, M + Na]+ [*m/z* 215.0315; 237.0135]) accumulated in the crude extract (Fig. S3). Furthermore, no 5dR formation was observed in the crude extracts (Fig. S4). With this, we exclude a spontaneous hydrolysis of 5dR-1P, which is in accordance to the literature, where 5dR-1P is reported to be metabolically stable (29).

### 5dR-1P is dephosphorylated by a specific phosphatase

**Figure 8:**
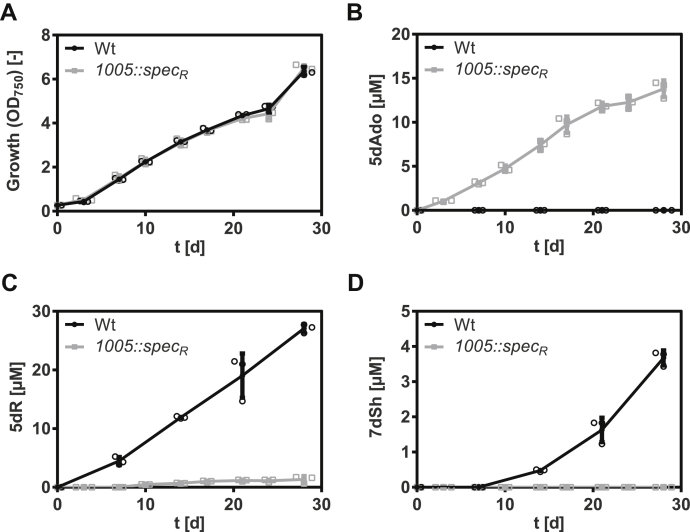
**5-Deoxyribose 1-phosphate (5dR-1P) is dephosphorylated by a phosphatase from the haloacid dehalogenase hydrolase superfamily (*Synpcc7942_1005*, Enzyme Commission number: 3.1.3.10).** Growth (*A*), concentrations of 5-deoxyadenosine (5dAdo) (*B*), 5-deoxyribose (5dR) (*C*), and 7-deoxy-sedoheptulose (7dSh) (*D*) in the supernatant of *Synechococcus elongatus* wildtype (*black dots*) or *1005*::*spec*_*R*_ mutant (*gray squares*). All cultures were aerated with air supplemented with 2% CO_2_. Note the different values of the *y*-axis. Values shown in the graphs represent mean and standard deviation of three biological replicates.

As 5dR-1P is metabolically stable, we assumed that for 5dR formation, a specific phosphatase must be involved. To identify this phosphatase, we analyzed the genome of *S. elongatus* regarding the presence of phosphoric monoester hydrolases (Table S3). *Synpcc7942_1005*, annotated as glucose-1-phosphatase, belonging to the haloacid dehalogenase (HAD)–like hydrolase superfamily subfamily IA (30, 31), seemed a promising candidate as only *S. elongatus* and *Synechococcus* sp. PCC 6301, which both produce larger amounts of 5dR/7dSh, possess a homologous gene. The other cyanobacteria mentioned previously do not possess it. Furthermore, phosphatases from the HAD-like hydrolase superfamily are known to be promiscuous enzymes dephosphorylating various phosphate sugars (32, 33). To examine whether this gene is essential for 5dR-1P dephosphorylation and thereby 5dR/7dSh synthesis, a corresponding mutant was created by replacing the *Synpcc7942_1005* gene with a spectinomycin resistance cassette (*S. elongatus Synpcc7942*_*1005*::*spec*_*R*_). Polar effects because of the insertion of the antibiotic resistance cassette were excluded by monitoring the expression of the genes with a semiquantitative RT-PCR (Fig. S2). Under 5dR/7dSh production conditions, the mutant grew like the wildtype (Fig. 8*A*). The wildtype excreted 5dR and 7dSh, whereas the mutant only excreted trace amounts of 5dR and not 7dSh (Fig. 8, *C* and *D*). Instead, the mutant excreted 5dAdo, which was never detected in the supernatant of the wildtype (Fig. 8*B*). This clearly shows that the gene product of *Synpcc7942_1005* is the major enzyme for the dephosphorylation of 5dR-1P. However, since in the mutant, small quantities of 5dR were detectable, other phosphatases may also contribute to minor 5dR-1P dephosphorylation. In agreement with this, *Synechoccoccus* sp. PCC 7002, which does not possess a homolog of *Synpcc7942_1005*, also excreted minor amounts of 5dR and 7dSh.

### 5dR/7dSh producers possess complete MSP gene clusters

By analyzing the genomes of all examined cyanobacteria in this study, it turned out that those strains that do not produce 5dR and 7dSh only possess annotated genes for the first two reactions of the MSP (*mtnP* and *mtnA*), whereas the producer strains possess annotated genes for the whole MSP pathway (Table S1). This suggests that the 5dR/7dSh producers might be able to rescue 5dAdo also *via* the DHAP shunt, as the third enzyme of the MSP, MTRu-1P-dehydratase (MtnB), can also act as a promiscuous aldolase, when no specific class II aldolase, as DrdA (*B. thuringiensis*) or Ald2 (*R. rubrum*), is present (13, 18). None of the analyzed strains possess an Ald2 homolog; therefore, the 5dR/7dSh nonproducer strains must employ another pathway of 5dAdo salvage.

With this, it appears that the pathway for 5dR and 7dS synthesis is clarified and based on the activity of seemingly promiscuous enzymes, which together catalyze the specific synthesis of the bioactive sugars. Moreover, as suggested by the CO_2_-promoted synthesis, the cells are apparently able to tune the metabolic flow in this pathway in response to environmental conditions.

## Discussion

Radical SAM enzymes are important enzymes in all domains of life (2). A byproduct of the activity of these enzymes is 5dAdo (3). Its accumulation inhibits the activity of the radical SAM enzymes themselves (9, 10, 11, 12). Therefore, 5dAdo salvage pathways are essential. In this study, we showed that the unicellular cyanobacterium *S. elongatus* PCC 7942 has a special salvage route for 5dAdo, which was never reported before (Fig. 1*A*). We show that 5dAdo salvage can be achieved by the excretion of 5-deoxyribose and 7-deoxysedoheptulose. 5dR as a product of 5dAdo cleavage was postulated (8, 17) or observed before but only in *in vitro* assays (9, 17). 5dR excretion was suggested as a detoxification strategy for organisms that do not possess a specific gene cluster for 5dAdo salvage (18) (analogous to MTR excretion in *E. coli*, which does not possess a complete MSP (34, 35)). Therefore, 5dR accumulation in the supernatant of *S. elongatus* as an *in vivo* phenomenon was first reported by our previous publication (20) and here identified as a result of 5dAdo salvage.

We propose the following model for a possible 5dAdo salvage route in *S. elongatus* by the activity of promiscuous enzymes leading to the synthesis of the bioactive deoxy-sugars 5dR and 7dSh (Fig. 1*A*). In brief, 5dAdo is processed by the promiscuous MtnP into 5dR-1P. Under elevated CO_2_ conditions, this molecule is dephosphorylated to 5dR by the presumably promiscuous phosphatase, the *Synpcc7942_1005* gene product, to 5dR, part of which is excreted and further metabolized by the activity of a promiscuous transketolase to 7dSh, which is also excreted to avoid the inhibition of the shikimate pathway (20). The producer strain tolerates high concentrations of 7dSh (Fig. 3), whereas other strains such as *A. variabilis* are highly sensitive toward 7dSh treatment (20), implying that 7dSh is a potent allelopathic inhibitor.

Although most bacteria possess the enzymes for a two-step reaction of 5dAdo cleavage (MtnN and MTR kinase) (36, 37), all examined cyanobacteria possess an MtnP (Table S1), which is normally present in eukaryotes (except for plants). The phenotype of the insertion mutant (*mtnP*::*spec*_*R*_), which excretes 5dAdo instead of 5dR/7dSh, demonstrates that 5dR and 7dSh are products of 5dAdo salvage (Fig. 6). The 5dAdo salvage routes previously reported suggest that the phosphorylation of 5dR or the 5dR moiety of 5dAdo is essential to further metabolize the molecules *via* specific enzymes or by promiscuous activity of the enzymes of the MSP (13, 17, 18). By contrast, in *S. elongatus*, 5dR-1P is dephosphorylated to 5dR or further processed to 7dSh to yield bioactive secreted metabolites. Our data imply that the dephosphorylation of 5dR-1P is not because of spontaneous hydrolysis but is mainly conducted by the gene product of *Synpcc7942*_*1005* (Fig. 8). *Synpcc7942*_*1005* belongs to Mg^2+^-dependent class IA HAD-like hydrolase superfamily (31) and is annotated as a glucose-1-phosphatase, which catalyzes the dephosphorylation of glucose 1-phosphate (38). As these phosphatases can also exhibit phytase activity (39, 40), we assume that the gene product of *Synpcc7942*_*1005* might also exhibit promiscuous activity, including 5dR-1P dephosphorylation. The dephosphorylation of a similar molecule (5-fluoro-5-deoxyribose 1-phosphate) by a specific phosphoesterase (FdrA) is also conducted by *Streptomyces* sp. MA37 during the production of a specific secondary fluorometabolite (41) (Fig. S5).

In later growth phases, part of 5dR is transformed into 7dSh, which is then also immediately excreted into the supernatant (Figs. 2*C* and 4, *C* and *E*). In our previous work, we showed that the affinity of *S. elongatus* transketolase for 5dR is 100-fold lower than for the natural substrate d-ribose-5-phosphate (20). This is in accordance with the fact that 7dSh is only formed when relatively high extracellular 5dR concentrations are reached (either in later growth phases or because of the addition of externally added 5dR; note that 5dR is continuously imported and exported). Furthermore, only one-tenth of ^13^C_5_-5dR is converted into ^13^C_5_-7dSh. 7dSh formation from 5dR is therefore an impressive example how a more potent “derivative” (7dSh) is formed by promiscuous enzyme activity. Interestingly, a promiscuous transketolase reaction was also suggested in later steps of anaerobic 5dAdo salvage in *M. jannaschii*, in which 5dRu-1P is cleaved into lactaldehyde and methylglyoxal (19). As our analysis showed, *S. setonensis* (not yet sequenced) accumulates much higher concentrations of 7dSh in the supernatant than *S. elongatus* but no 5dR at all. If *S. setonensis* synthesizes 7dSh *via* the same pathway than *S. elongatus*, the complete conversion of 5dR could be due to a more specific transketolase.

In high concentrations, 5dR exhibited toxicity toward the producer strain (Fig. 3). 5dR toxicity was also reported in *B. thuringiensis* (18), but the intracellular target is not yet known. Therefore, *S. elongatus* has to steadily excrete 5dR into the supernatant to avoid intracellular toxicity. Because ^13^C_5_-5dR was taken up at the same time as unlabeled 5dR was excreted (Fig. 4, *B* and *D*), specific transport systems have to be present, which are probably essential for the survival of the producer strain.

5dAdo salvage *via* 5dR and 7dSh excretion was only observed when cultures were aerated with air supplemented with 2% CO_2_ (Fig. 2, *B* and *C*). Since equal amounts of 5dAdo were formed under ambient CO_2_ as under high CO_2_ conditions (Fig. 6*E*), we assumed that under ambient conditions, 5dAdo salvage is conducted *via* (an)other pathway(s). The occurrence of (an) additional 5dAdo salvage pathway(s) in *S. elongatus* is underlined by the fact that 5dAdo is not completely metabolized into 5dR/7dSh even under high CO_2_ conditions (Fig. 5). Because *S. elongatus* and the other 5dR/7dSh producers are equipped with the enzymes for the whole MSP (Table S1), we hypothesize that 5dAdo can be also metabolized *via* promiscuous activity of the enzymes of the MSP *via* the “DHAP-shunt” resulting in the formation of DHAP and acetaldehyde (Fig. 1, *A* and *C*) as suggested for organisms that do not possess a specific gene cluster for 5dAdo salvage (13, 17, 18). The formation of MTA, the starting molecule of the MSP, is almost identical under atmospheric and high carbon conditions (Fig. 6*E*). This indicates that 5dAdo salvage *via* 5dR/7dSh excretion under high CO_2_ conditions is not triggered by an increased demand of MTA salvage. It is known that intracellular CO_2_/HCO_3_^−^ (C_i_) exhibits regulatory functions at the metabolic and transcriptomic levels (42), and it is known to regulate virulence and toxin production in pathogens, for example, in *Vibrio cholerae* (43). In particular, cyanobacteria strongly respond to the ambient C_i_ supply by a multitude of metabolic adaptations such as carbon concentrating mechanisms (44) and the synthesis of cAMP (45). As we hypothesize that the fate of 5dAdo is a regulated process, we assume that the dephosphorylation of 5dR and the subsequent formation of 7dSh molecules is not an “accident”. They are rather purposely formed metabolites, which however derive from toxic byproducts of the primary metabolism. The regulation how 5dAdo is directed toward 5dR/7dSh formation has to be further investigated.

With 18 radical SAM enzymes (Table S2), *S. elongatus* only possesses a relatively small number of radical SAM enzymes compared with other prokaryotes (*B. thuringiensis*: 15; other Firmicutes: more than 40 (18); *R. rubrum*: 25; and *M. jannaschii*: 30 (13)). Probably the most important radical SAM enzymes under the cultivation conditions applied here are involved in cofactor biosynthesis and presumably equally important under ambient or high carbon conditions resulting in the unaltered 5dAdo formation.

7dSh can inhibit the growth of not only other cyanobacteria but also plants and was therefore suggested to be an allelopathic inhibitor by inhibiting the dehydroquinate synthase, the second enzyme of the shikimate pathway (20). In addition, 5dR is toxic for various organisms (Fig. 3; (18)). Despite the low concentrations of 5dR/7dSh observed under laboratory conditions, it is imaginable that excretion of 5dR and 7dSh plays a role in protecting the ecological niche of the producer strains. 7dSh is a more potent inhibitor, for example, for *A. variabilis* than for the producer strain. A bactericidal effect for *A. variabilis* was observed at concentrations of 13 μM 7dSh (20), whereas *S. elongatus* is affected by 100 μM (Fig. 3). Although it is not obvious from isolated *in vitro* studies, we speculate that 7dSh might play a role in niche competition. In its natural environment, *S. elongatus* can live planktonically, but it is also able to form biofilms or microbial mats, which also contain the colonization of caves and humid stonewalls (46, 47, 48, 49, 50). In the latter habitats, the dilution of excreted compounds is prevented, and therefore, the activity of 7dSh as an allelopathic inhibitor is imaginable. In addition, cyanobacteria tend to excrete exopolysaccharides in biofilms (51), which can be used as a carbon source by heterotrophic members of the microbial community, thereby causing locally elevated CO_2_ concentrations. This could lead to a local enrichment of 5dR and 7dSh, thereby providing a growth advantage to the producer strains protecting their niches against competing microalgae.

5dAdo salvage is a less noticeable and overlooked research topic in comparison to methionine salvage from MTA. Hence, it should be further investigated above all because 5dAdo is present in all domains of life, whereas MTA is only produced by specific organisms. Since 5dAdo disposal pathways seem to differ from species to species, our findings suggest that other noncanonical 5dAdo salvage pathways may exist, encouraging the search of “cryptic” metabolites derived from this pathway.

Overall, this study shows a unique example of a synthesis pathway of bioactive molecules solely catalyzed by promiscuous enzymes of primary metabolism, which challenges the current view on the synthesis of bioactive molecules. The involvement of enzyme multifunctionality extends the range of possible bioactive compounds far beyond what can be predicted from standard genome mining biased for secondary metabolite gene clusters.

## Experimental procedures

### Cultivation

*S. elongatus* PCC 7942 was cultivated under photoautotrophic conditions in BG11 medium (52) supplemented with 5 mM NaHCO_3_. Precultures were cultivated in shaking flasks at 30 to 50 μE at 125 rpm (27 °C). Main cultures were cultivated in 500 to 700 ml BG11 at 27 °C in flasks that were either aerated with air or air supplemented with 2% CO_2_. For this purpose, cultures were inoculated with an optical density at 750 nm of 0.2 to 0.5 and then cultivated for the first 3 days at 10 μE (Lumilux de Lux; Daylight; Osram). Later, the light intensity was set to around 30 μE. Growth was determined by measuring the absorbance at 750 nm (Specord 205; Analytik Jena). For feeding experiments, the cultures were supplemented at the beginning of the cultivation with 5dR, [U-^13^C_5_]-5dR, or 5dAdo (Carbosynth Ltd) at the respective concentrations (see Results section). The other cyanobacterial strains (*Synechococcus* sp. PCC 6301, *Synechococcus* sp. PCC 6312, *Synechococcus* sp. PCC 7502, *Synechocystis* sp. PCC 6803, *A. variabilis* ATCC 29413, *N. punctiforme* ATCC 29133, and *Anabaena* sp. PCC 7120) were cultivated as described previously. *Synechococcus* sp. PCC 7002 was cultivated in a 1:1 mixture of BG11 and ASN III + vitamin B_12_ (10 μg/ml) (52).

*S. setonensis* SF666 was cultivated for 7 days as described in our previous work (20).

### Synthesis of 5-deoxyribose and 7-deoxysedoheptulose

5dR and [U-^13^C_5_]-5dR **5** were synthesized in a four-step synthesis based on the literature (53, 54) with an additional optimization. All synthetic intermediates shown in the reaction scheme (Fig. S6) were verified by TLC, MS, and NMR. Detailed data for the ^13^C-labeled compounds are presented in the Supporting Information. The synthesis starts with the reaction of d-ribose (Sigma) or [U-^13^C_5_]-d-ribose **1** (500.1 mg, 3.22 mmol; Eurisotop) in a 4:1 mixture of acetone:methanol with SnCl_2_×2 H_2_O (1 eq) and catalytic amounts of concentrated H_2_SO_4_ at 45 °C for 20 h. After cooling to room temperature, the mixture was filtered, neutralized with NaHCO_3_ solution, once again filtered, and the organic solvent was evaporated. The remaining aqueous solution was extracted with ethylacetate, dried over Na_2_SO_4_, and evaporated *in vacuo* to yield the acetonide-protected ribose **2** as a colorless oil (399.7 mg, 1.91 mmol, 59%).

Envisaging the following deoxygenation reaction, the protected pentose **2** (399.7 mg, 1.91 mmol) was diluted in dilated cardiomyopathy with addition of triethylamine (2.5 eq). After cooling on ice, mesylchloride (2.5 eq) was slowly added and then stirred for 5 h on ice. The reaction mixture was washed with 1 N HCl, ultrapure water, NaHCO_3_ solution, NaCl solution, and again with ultrapure water. The organic solvent was dried over Na_2_SO_4_ and evaporated *in vacuo* to give **3** as an yellowish oil (556.5 mg, 1.97 mmol, 103%, mesylchloride as impurity), which becomes crystalline at 4 °C.

For the reduction as the third step **3** (556.1 mg, 1.91 mmol, maximum educt amount) was diluted in dimethyl sulfoxide. After cooling on ice, NaBH_4_ (5 eq) was added slowly. Afterward, the reaction mixture was heated slowly to 85 °C and reacting for 12 h. After cooling on ice, 5% AcOH was added to quench remaining NaBH_4_. The aqueous solution was extracted with dilated cardiomyopathy, washed with ultrapure water, dried over Na_2_SO_4_, and evaporated *in vacuo* (40 °C, 750 mbar) to get **4** as a colorless oil (357.7 mg, 1.85 mmol, 86%).

Deprotecting to the target **5** was achieved by diluting the acetonide-protected ω-deoxy-sugar **4** (357.7 mg, 1.85 mmol) in 0.04 N H_2_SO_4_ and heating to 85 °C for 3 h. After cooling to room temperature, the reaction mixture was neutralized with NaHCO_3_ solution and evaporated by lyophilization. The final product was first purified by medium-pressure liquid chromatography (gradient: start CHCl_3_:MeOH 10:0; end CHCl_3_:MeOH 7:3) and HPLC (column: HiPlexCa, 85 °C, 250 × 10.7 mm, 1.5 ml/min, solvent: ultrapure water) to get [U-^13^C_5_]-5-deoxy-d-ribofuranose **5** as a colorless oil (115.7 mg, 1.12 mmol, 61%).

7dSh or [3,4,5,6,7-^13^C_5_]-7dSh was synthesized in a transketolase-based reaction with 5dR or [U-^13^C_5_]-5dR as substrate as described in our previous publication (20) with slight modifications. The reaction was performed in water instead of Hepes buffer to ensure an enhanced stability of hydroxypyruvate (very unstable in Hepes (55)). The reaction was performed for 7 days, and fresh hydroxypyruvate was added every day. Purification was done as described for 5dR.

### Construction of insertion mutants

To create an insertion mutant of the 5-methylthioadenosine phosphorylase (Enzyme Commission number: 2.4.2.28, MtnP, *Synpcc7942_0932*) and glucose-1-phosphatase (Enzyme Commission number: 3.1.3.10, *Synpcc7942*_*1005*) in *S. elongatus* PCC 7942, a spectinomycin resistance cassette was introduced inside the respective gene. An integrative plasmid was constructed in *E. coli* and then transformed into *S. elongatus*. For this purpose, flanking regions on both sides of the respective gene were amplified from *S. elongatus* colonies with primers adding an overlapping fragment (46_0923_up_fw, 47_0923_up_rev; 48_Δ0923_down_fw and 49_0923_down_rev for *Synpcc7942*_*0923*::*spec*_*R*_; 85_1005_up_fw, 86_1005_up_rev; 87_1005_down_fw and 88_1005_down_rev for *Synpcc7942*_*1005*::*spec*_*R*_; sequences are shown in Table S4). The spectinomycin resistance cassette was amplified with primers 32_Spec_fw and 33_Spec_rev. All PCR amplification products were introduced into a pUC19 vector cut with XbaI and PstI by using Gibson assembly (56). The plasmid was verified by Sanger sequencing (Eurofins Genomics). The plasmid was then transformed into *S. elongatus* using natural competence as described elsewhere (57). Segregation was confirmed by colony PCR (50_0923_rev_seg and 51_0923_fw_seg for *Synpcc7942*_*0923*::*spec*_*R*_; 85_1005_up_fw and 88_1005_down_rev for *Synpcc7942*_*1005*::*spec*_*R*_). Precultures of these strains, in the following named as *S. elongatus mtnP*::*spec*_*R*_ or *S. elongatus 1005*::*spec*_*R*_ were cultivated in the presence of 20 μg/ml spectinomycin, main cultures without antibiotic.

### Quantification of metabolites in the culture supernatant *via* GC–MS

Culture supernatant was collected by centrifugation of 1.5 ml culture (16.000*g*, 10 min, 4 °C). About 200 μl of the supernatant was immediately frozen on liquid nitrogen and stored at −80 °C. Before extraction, the supernatant was lyophilized. For intracellular measurements, the cell pellets were also frozen in liquid nitrogen. Samples were extracted as described in the literature (58) with slight modifications: 700 μl of ice-cold extraction solution (CHCl_3_/MeOH/H_2_O in a ratio of 1/2.5/0.5 v/v/v) were either added to 200 μl of the lyophilized supernatant or to cell pellets. Samples were homogenized by vortexing, ultrasonic bath (Bandelin, Sonorex) treatment (10 min), and shaking (10 min, 1.000 rpm). After that, the samples were cooled on ice for 5 min and then centrifuged (10 min, 16.000*g*, 4 °C). The supernatant was transferred into a new reaction tube. The pellet was again extracted with 300 μl extraction solvent as described before. The supernatants were pooled, and 300 μl ice-cold water was added for phase separation. The samples were vortexed, incubated on ice (5 min), and then centrifuged (10 min, 16.000*g*, 4 °C). About 900 μl of the upper polar phase was transferred into a new 2 ml reaction tube and dried in a vacuum concentrator (Eppendorf, Concentrator plus, mode: V-AQ, 30 °C) for approximately 4.5 h. The samples were immediately closed and then derivatized as described in the literature (59) with slight modifications. Therefore, the pellets were resolved in 60 μl methoxylamine hydrochloride (Acros Organics) in pyridine (anhydrous, Sigma–Aldrich) (20 mg/ml), homogenized by vortexing, a treatment in an ultrasonic bath (15 min), and an incubation at 30 °C on a shaker (1.400 rpm) for 1.5 h. After that, 80 μl *N*-methyl-*N*-(trimethylsilyl)trifluoroacetamide (Macherey-Nagel) was added, and the samples were incubated at 37 °C for 30 min (1.200 rpm). The samples were centrifuged (16.000*g*, 2 min), and 120 μl was transferred into a glass vial with microinsert. The samples were stored at room temperature for 2 h before GC–MS measurement.

GC–MS measurements were performed on a Shimadzu GC–MS TQ 8040 (injector: AOC-20i; sampler: AOC-20s) with an SH-Rxi-5Sil-MS column (Restek; 30 m, 0.25 mm ID, 0.25 μm). For GC measurement, the initial oven temperature was set to 60 °C for 3 min. After that, the temperature was increased by 10 °C/min up to 320 °C, which was then held for 10 min. The GC–MS interface temperature was set to 280 °C, and the ion source was heated to 200 °C. The carrier gas flow (helium) was 1.28 ml/min. The injection was performed in split mode 1:10. The mass spectrometer was operated in exposure index mode. Metabolites were detected in multiple reaction monitoring mode. Quantification of the metabolites was performed with a calibration curve of the respective substances (5dAdo, 5dR, 7dSh, ^13^C_5_-5dR, and ^13^C_5_-7dSh). The recovery efficiency of exogenously added standards (^13^C_5_-5dR and ^13^C_5_-7dSh) during the extraction of the cell pellets, as well as during extraction of the supernatant, is shown in the Supporting Information (Fig. S7 and Supporting text).

### Quantification of MTA and 5dAdo

For the quantification of MTA and 5dAdo (Fig. 6*E*), 25 μl of culture supernatant was mixed with 75 μl aqueous solution of 20% MeOH (v/v) + 0.1% (v/v) formic acid. Samples were analyzed on an LC–HR-MS system (Dionex Ultimate 3000 HPLC system coupled to maXis 4G ESI-QTOF mass spectrometer). 5dAdo and MTA were separated on a C18 column with an MeOH/H_2_O gradient (10%–100% in 20 min). The concentration was calculated from peak areas of extracted ion chromatograms of a calibration curve of the respective standards (MTA was obtained from Cayman Chemicals).

### Crude extract assays

Crude extract assays were performed by harvesting 10 ml of the cultures after 14 days of cultivation (air supplemented with 2% CO_2_) by centrifugation (3.200*g*, 10 min, 4 °C). The supernatant was discarded, and the pellet was washed with 10 ml fresh medium. The pellet was resuspended in 2.5 ml lysis buffer (25 mM Hepes pH 7.5, 50 mM KCl, 1 mM DTT). Cell disruption was performed in a FastPrep-24 instrument (MP Biomedicals, 5 m/s, 20 s, 3× with 5 min break) by adding glass beads (⌀ = 0.1–0.11 mm). Cell debris was removed by centrifugation (25.000*g*, 10 min, 4 °C). About 200 μl of the extract was either used alone or supplemented with 5dAdo (final concentration: 250 μM) or in combination with potassium phosphate buffer pH 7.5 (final concentration: 10 mM). The extracts were incubated at 28 °C for 7 h, frozen in liquid nitrogen, and lyophilized. About 100 μl MeOH was added, and the samples were homogenized and centrifuged. About 50 μl was applied on a TLC plate (ALUGRAM Xtra SIL G UV_254_; Macherey-Nagel). For the mobile phase, CHCl_3_/MeOH in a ratio of 9:5 (v/v) with 1% (v) formic acid was used. Visualization was performed at 254 nm (Fig. 7) or spraying with anisaldehyde (Fig. S4).

### Bioinformatics

Annotations of the different genes were obtained from the Kyoto Encyclopedia of Genes and Genomes database (60). Also, radical SAM enzyme (pf: Radical_SAM, PF04055) search was done in Kyoto Encyclopedia of Genes and Genomes database. Searching for homologous genes was performed by using BlastP (BLOSUM 62). Searching for Ald2 homologs, *R. rubrum* protein sequence (rru:Rru_A0359) was used as a query sequence, and an e value <10e-20 was used for positive results.

## Data availability

All data are presented in the article, in the supporting information, or are available upon request (please contact: Karl Forchhammer, karl.forchhammer@uni-tuebingen.de).

## Supporting information

This article contains supporting information (15, 18, 41, 53, 54, 60, 61).

## Conflict of interest

The authors declare that they have no conflicts of interest with the contents of this article.
