# Supplementary material for: A bioactive molecule made by unusual salvage of radical SAM enzyme byproduct 5-deoxyadenosine blurs the boundary of primary and secondary metabolism
Source: J Biol Chem. 2021 Mar 31;296:100621. doi: 10.1016/j.jbc.2021.100621 (PMC8102628; doi:10.1016/j.jbc.2021.100621)
Supplement: Supporting data [file mmc1.pdf]

Supporting information for:

A bioactive molecule made by unusual salvage of radical SAM enzyme by-product 5-deoxyadenosine blurs the boundary of primary and secondary metabolism

Johanna Rapp, Pascal Rath, Joachim Kilian, Klaus Brilisauer, Stephanie Grond, Karl Forchhammer

Results

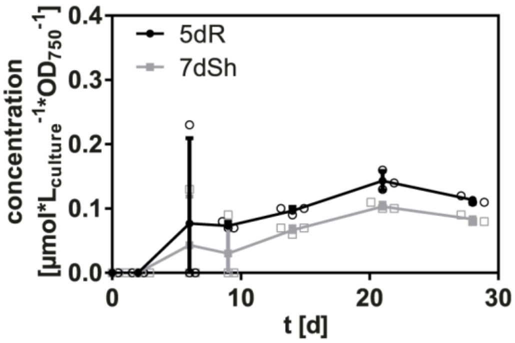

Figure S1: Only small intracellular 5dR and 7dSh accumulation in *S. elongatus*. Concentration of 5dR (black dots) and 7dSh (grey squares) in *S. elongatus* cells [µmol*$L^{-1}_{culture}$*OD_{750}^{-1}] aerated with air supplemented with 2 % CO_2.

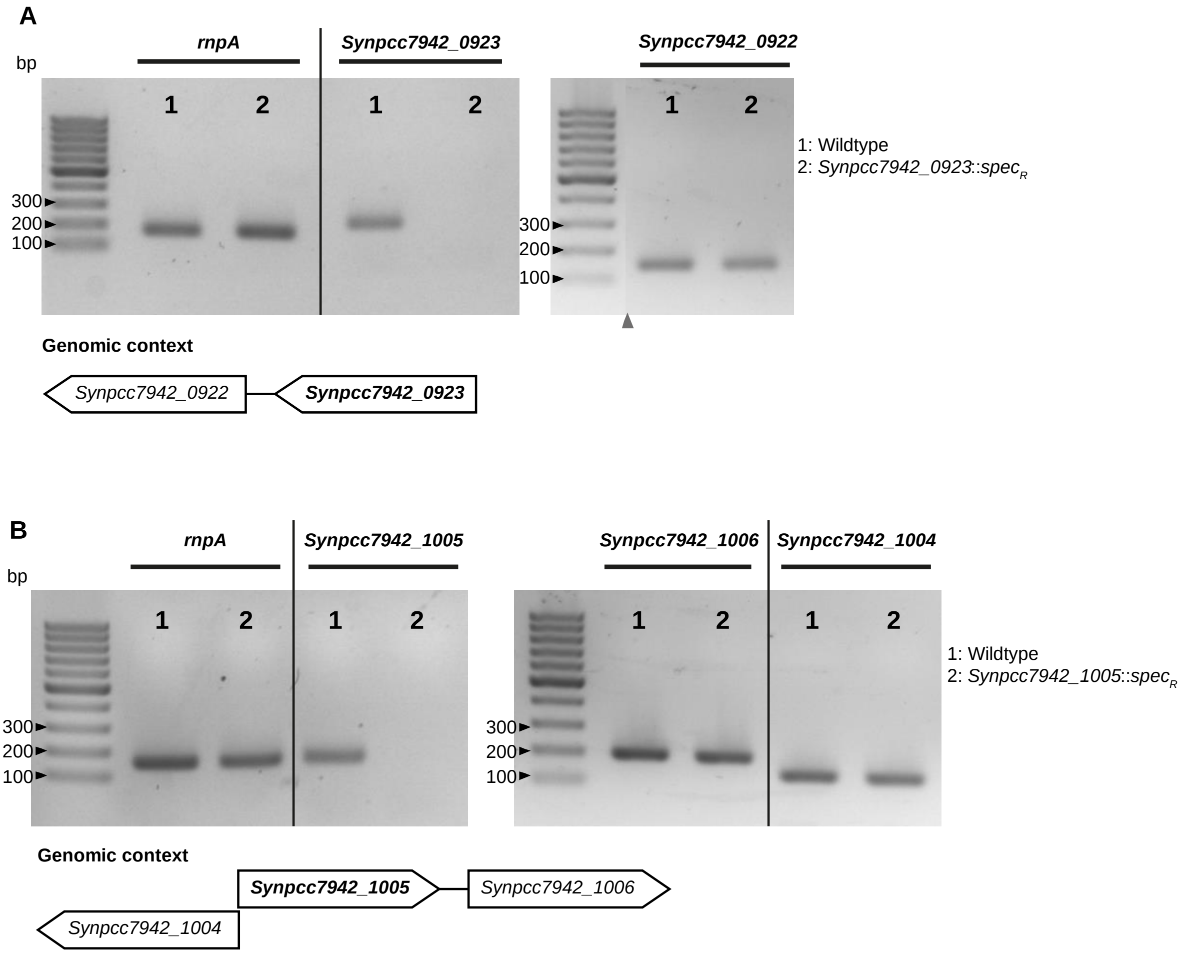

Figure S2: Gene expression of selected genes in *S. elongatus* wildtype and in the insertion mutants *Synpcc7942_0923*::*spec*$_R$ (A) and *Synpcc7942_1005*::*spec*$_R$ (B) determined by semi-quantitative RT-PCR and genomic context of the deleted genes. RNA was extracted from the cell pellets, converted into cDNA by using reverse transcriptase. Gene expression was determined by amplifying the deleted gene and down- or upstream genes by using gene-specific primers (see Table S4) resulting in fragments of 100-200 bp. Amplified fragments were analysed by using agarose gel-electrophoresis. Splice borders are labelled with grey triangle, but marker and samples were run on the same gel. The expression of the house-keeping gene *rnpA* served as a control.

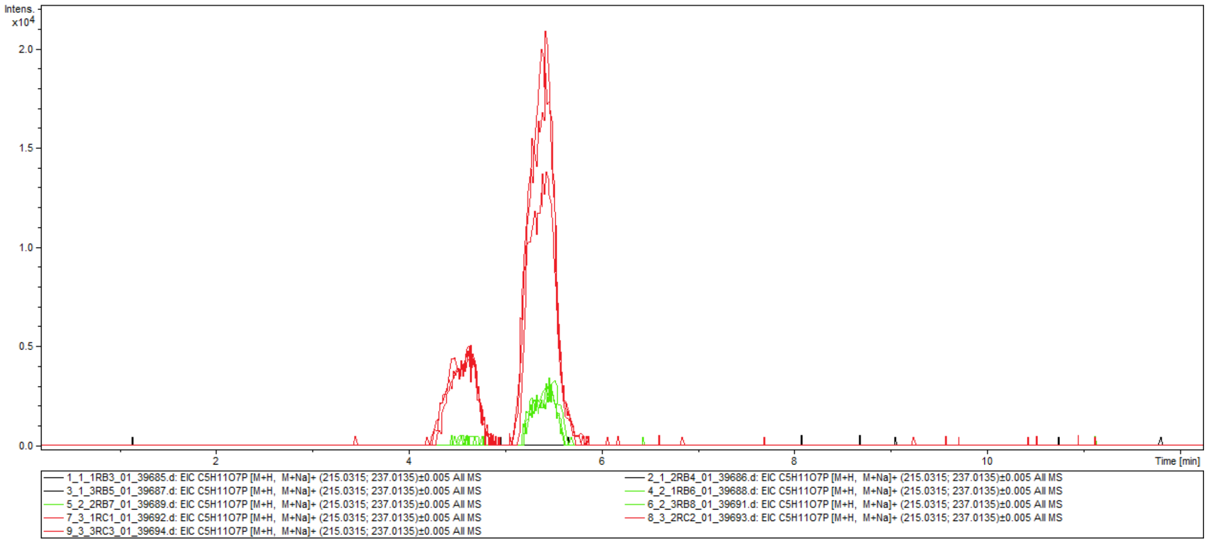

Figure S3: 5dR-1P accumulates in crude extracts of *S. elongatus* that were incubated in the presence of phosphate. Accumulation of 5dR-1P shown as extracted ion chromatogram [M+H, M+Na]+ (*m/z* 215.0315; *m/z* 237.0135) in crude extracts of *S. elongatus*, (Red – with 5dAdo+potassium phosphate buffer (PPB); green – with 5dAdo, no PPB; black – without 5dAdo, PPB). Three independent replicates are shown for each treatment. One part of the samples of the crude extract assays was analysed via high resolution LC-MS (C18 Gemini, solvent A: ACN+0.1 %TFA, solvent B: H_2O, 1% - 20% B in 20 min, Maxis 4G ESI-QTOF).

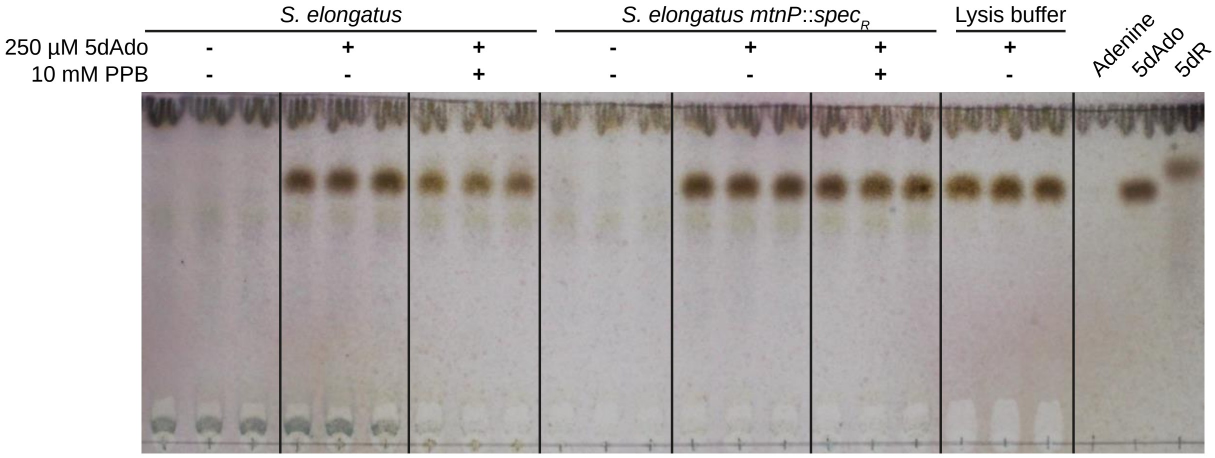

Figure S4: 5dR does not accumulate in crude extracts which were incubated with 5dAdo. Crude extracts from *S. elongatus* or *S. elongatus mtnP*::*spec*$_R$ were incubated with 5dAdo in the presence or absence of potassium phosphate buffer (PPB) and then analysed via thin layer chromatography (TLC). TLC plate from Figure 7 (main text) was sprayed with anisaldehyde after UV-visualisation. Pure adenine, 5dAdo and 5dR were used as standards (right). Adenine is only visible with UV-visualisation (see Figure 7, main text). Three independent replicates are shown for each condition.

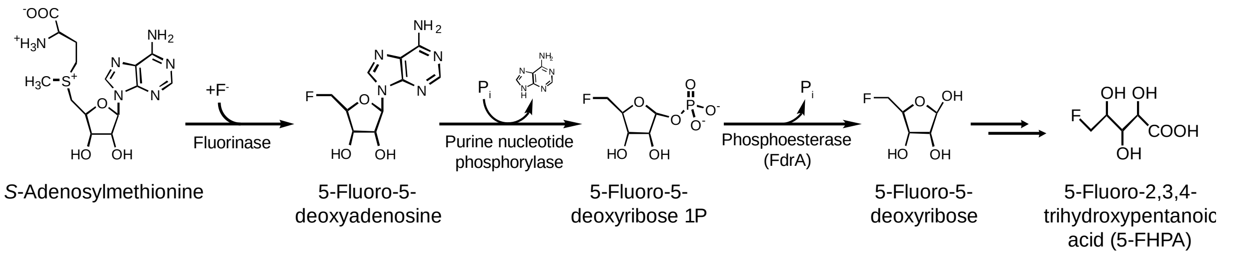

Figure S5: Biosynthesis of the fluoro-metabolite 5-FHPA in *Streptomyces* sp. MA37 (modified after (1)).

Table S1: Overview of MSP genes present in different cyanobacteria.

Gene name in *B. subtilis*		MtnN	MtnK	MtnP*	MtnA	MtnB	MtnC***	MtnX	MtnW	MtnD	MtnE
**Gene Product**		**MTA nucleosidase**	**MTR kinase**	**MTA phosphory-lase**	**MTR-1P isomerase**	**MTRu-1P dehydratase ****	**enolase/ phosphatase[*1]**	**phospha-tase[*2]**	**enolase[*3]**	**dioxygenase[*4]**	**amino transferase ******
Strain	Identifier	EC 3.2.2.9	EC 2.7.1.100	EC 2.4.2.28	EC 5.3.1.23	EC 4.2.1.109	EC 3.1.3.77	EC 3.1.3.87	EC 5.3.2.5	EC 1.13.11.53/54	EC 2.6.1.117
PCC 7942	syf: Synpcc7942_	-	-	0923	1992	1993	1994	0510	-	0608	2.6.1.-
PCC 6301	syc: syc	-	-	0619_d	2104_d	2103_c	2102_c	1010_d	-	0916_c	2.6.1.-
PCC 7002	syp: SYNPCC7002_	-	-	A0108	A2308	A0554	A0552	A0257	-	A0553	2.6.1.-
PCC 6803	syn:	-	-	sll0135	slr1938	-	-	-	-	-	2.6.1.-
PCC 7120	ana:	-	-	alr4054	all3566	-	-	-	-	all2724	2.6.1.-
PCC 7502	synp: Syn7502_	-	-	03055	00983	-	-	-	-	-	2.6.1.-
PCC 6312	syne: Syn6312_	-	-	2991	2219	-	-	-	-	-	2.6.1.-
ATCC 29431	ava:	-	-	Ava_1653	Ava_3544	-	-	-	-	Ava_4291	2.6.1.-
ATCC 29133	npu: Npun_	-	-	F6610	F5471	-	F4952	-	-	-	2.6.1.-

Gene abbreviations according to *B. subtilis* annotation in which the MSP was discovered (2, 3). Gene identifiers are referred to KEGG (4).
* not in *B. subtilis*
** For *A. thaliana* MTR-1P dehydratase (DEP1) moonlighting aldolase activity on 5-deoxyribulose 1-phosphate was shown (5).
*** not in *B. subtilis*. MtnC (EC 3.1.3.77) is a bifunctional enzyme, which has enolase and phosphatase activity. In *B. subtilis* this step is performed by two enzymes (MtnW: 2,3-diketo-5-methylthiopentyl-1-phosphate enolase (EC 5.3.2.5) and MtnX: 2-hydroxy-3-keto-5-methylthiopentenyl-1-phosphate phosphatase (EC 3.1.3.87).
**** aminotransferases are normally broad specificity enzymes (3)
[*1] 2,3-dioxomethio-pentane-1P enolase/phosphatase
[*2] 2-hydroxy-3-keto-5-methylthiopentenyl-1-phosphate phosphatase
[*3] 2,3-diketo-5-methylthiopentyl-1-phosphate enolase
[*4] 1,2-dihydroxy-3-keto-5-methylthiopentene dioxygenase

Table S2: Genes encoding for SAM radical enzymes in *S. elongatus* PCC 7942. KEGG genes were examined for the presence of the Pfam motif PF04055 (SAM_radical), which is a distinctive feature of radical SAM enzymes. Table shows accession number and annotations from KEGG. GenBank annotations are only shown if using another annotation.

Accesion No.	Annotation
Synpcc7942_0419	K01012 biotin synthase [EC:2.8.1.6]
Synpcc7942_0542	K03644 lipoyl synthase [EC:2.8.1.8]
Synpcc7942_0686	K11781 5-amino-6-(D-ribitylamino)uracil---L-tyrosine 4-hydroxyphenyl transferase [EC:2.5.1.147] | (GenBank) FO synthase subunit 2
Synpcc7942_0799	no KO assigned | (GenBank) Elongator protein 3
Synpcc7942_0838	no KO assigned | (GenBank) Elongator protein 3/MiaB/NifB
Synpcc7942_0877	no KO assigned | (GenBank) Elongator protein 3/MiaB/NifB
Synpcc7942_0892	K11780 7,8-didemethyl-8-hydroxy-5-deazariboflavin synthase [EC:4.3.1.32] | (GenBank) FO synthase subunit 1
Synpcc7942_0945	no KO assigned | (GenBank) conserved hypothetical protein
Synpcc7942_1229	K05936 precorrin-4/cobalt-precorrin-4 C11-methyltransferase [EC:2.1.1.133 2.1.1.271]
Synpcc7942_1282	K03639 GTP 3',8-cyclase [EC:4.1.99.22] | (GenBank) GTP cyclohydrolase subunit MoaA
Synpcc7942_1332	K10026 7-carboxy-7-deazaguanine synthase [EC:4.3.99.3] | (GenBank) conserved hypothetical protein
Synpcc7942_1507	K03644 lipoyl synthase [EC:2.8.1.8] | (GenBank) lipoic acid synthetase
Synpcc7942_1621	no KO assigned | (GenBank) Elongator protein 3/MiaB/NifB
Synpcc7942_1652	no KO assigned | (GenBank) Elongator protein 3/MiaB/NifB
Synpcc7942_1758	K06941 23S rRNA (adenine2503-C2)-methyltransferase [EC:2.1.1.192] | (GenBank) conserved hypothetical protein
Synpcc7942_2374	K06168 tRNA-2-methylthio-N6-dimethylallyladenosine synthase [EC:2.8.4.3] | (GenBank) tRNA-i(6)A37 thiotransferase enzyme MiaB
Synpcc7942_2382	no KO assigned | (GenBank) coproporphyrinogen III oxidase, anaerobic
Synpcc7942_2512	K14441 ribosomal protein S12 methylthiotransferase [EC:2.8.4.4] | (GenBank) Protein of unknown function UPF0004

Table S3: Genes encoding for phosphoric monoester hydrolases [EC: 3.1.3.-] in *S elongatus* PCC 7942. Table shows accession number and annotations from KEGG. GenBank annotations are only shown if using another annotation.

Accesion No.	Annotation
Synpcc7942_0173	K01082 3'(2'), 5'-bisphosphate nucleotidase [EC:3.1.3.7] | (GenBank) 3'-Phosphoadenosine 5'-phosphosulfate (PAPS) 3'-phosphatase-like
Synpcc7942_0463	K01104 protein-tyrosine phosphatase [EC:3.1.3.48] | (GenBank) protein tyrosine phosphatase
Synpcc7942_0485	K22305 phosphoserine phosphatase [EC:3.1.3.3] | (GenBank) phosphoglycerate mutase
Synpcc7942_0505	K11532 fructose-1,6-bisphosphatase II / sedoheptulose-1,7-bisphosphatase [EC:3.1.3.11 3.1.3.37]
Synpcc7942_0510	K08966 2-hydroxy-3-keto-5-methylthiopentenyl-1-phosphate phosphatase [EC:3.1.3.87]
Synpcc7942_0613	K08296 phosphohistidine phosphatase [EC:3.1.3.-] | (GenBank) phosphohistidine phosphatase, SixA
Synpcc7942_0693	K01091 phosphoglycolate phosphatase [EC:3.1.3.18] | (GenBank) conserved hypothetical protein
Synpcc7942_0791	K00974 tRNA nucleotidyltransferase (CCA-adding enzyme) [EC:2.7.7.72 3.1.3.- 3.1.4.-] | (GenBank) polyA polymerase
Synpcc7942_0965	K01082 3'(2'), 5'-bisphosphate nucleotidase [EC:3.1.3.7] | (GenBank) ammonium transporter protein Amt1-like
Synpcc7942_0976	K00974 tRNA nucleotidyltransferase (CCA-adding enzyme) [EC:2.7.7.72 3.1.3.- 3.1.4.-] | (GenBank) CBS
Synpcc7942_1005	K20866 glucose-1-phosphatase [EC:3.1.3.10] | (GenBank) HAD-superfamily hydrolase subfamily IA, variant 3
Synpcc7942_1130	K01090 protein phosphatase [EC:3.1.3.16] | (GenBank) protein serine/threonine phosphatase
Synpcc7942_1515	K01090 protein phosphatase [EC:3.1.3.16] | (GenBank) protein serine/threonine phosphatase
Synpcc7942_1553	K07053 3',5'-nucleoside bisphosphate phosphatase [EC:3.1.3.97] | (GenBank) Phosphoesterase PHP-like
Synpcc7942_1763	K01092 myo-inositol-1(or 4)-monophosphatase [EC:3.1.3.25] | (GenBank) inositol monophosphate family protein
Synpcc7942_1931	K07313 serine/threonine protein phosphatase 1 [EC:3.1.3.16] | (GenBank) probable serine/threonine protein phosphatase
Synpcc7942_1994	K09880 enolase-phosphatase E1 [EC:3.1.3.77] | (GenBank) 2,3-diketo-5-methylthio-1-phosphopentane phosphatase
Synpcc7942_2063	K03787 5'-nucleotidase [EC:3.1.3.5] | (GenBank) exopolyphosphatase / 5'-nucleotidase / 3'-nucleotidase
Synpcc7942_2076	K06949 ribosome biogenesis GTPase / thiamine phosphate phosphatase [EC:3.6.1.- 3.1.3.100] | (GenBank) GTPase EngC
Synpcc7942_2288	K03270 3-deoxy-D-manno-octulosonate 8-phosphate phosphatase (KDO 8-P phosphatase) [EC:3.1.3.45] | (GenBank) Phosphatase kdsC
Synpcc7942_2335	K03841 fructose-1,6-bisphosphatase I [EC:3.1.3.11] | (GenBank) D-fructose 1,6-bisphosphatase
Synpcc7942_2473	K07315 phosphoserine phosphatase RsbU/P [EC:3.1.3.3] | (GenBank) serine phosphatase
Synpcc7942_2582	K01092 myo-inositol-1(or 4)-monophosphatase [EC:3.1.3.25]
Synpcc7942_2589	K05979 2-phosphosulfolactate phosphatase [EC:3.1.3.71]
Synpcc7942_2613	K01091 phosphoglycolate phosphatase [EC:3.1.3.18] | (GenBank) HAD-superfamily hydrolase subfamily IA

Material & Methods

Table S4: Oligonucleotides for PCR amplification (G), sequencing (S) and semi-quantitative RT-PCR (qPCR). Overlapping fragments for Gibson cloning are labelled in bold.

Name	Sequence (5' → 3')
46_0923_up_fw (G)	**AGCTCGGTACCCGGGGATCCT**GGCCTCTACCGAATGGAAGC
47_0923_up_rev (G)	**CTGCGTTCGGTCAAGAGCT**TTGCCAAAGAAGGTCGAAGG
32_Spec_fw (G)	GAGCTCTTGACCGAACGCAG
33_Spec_rev (G)	TTATTTGCCGACTACCTTGGTGATCTC
48_0923_down_fw (G)	**GAGATCACCAAGGTAGTCGGCAAATAA**CCCAGATTATCGGCATGACC
49_0923_down_rev (G)	**ACGCCAAGCTTGCATGCCTGCA**TGCAGGAGTAGTGCCAAACG
1064_pUC19_fw (S)	TGCTGCAAGGCGATTAAGTTGGG
1065_pUC19_rev (S)	CGACAGGTTTCCCGACTGGAAAG
50_0923_rev_seg (S)	CTAGTCGACCCGCTTCAACC
51_0923_fw_seg (S)	GCCACCAAGGATCCCAGATG
85_1005_up_fw	**AGCTCGGTACCCGGGGATCCT**TACAACCGCCTCAAGTGC
86_1005_up_rev	**CTGCGTTCGGTCAAGAGCT**ATGGAGCGTCCCGAAGTAAG
87_1005_down_fw	**GAGATCACCAAGGTAGTCGGCAAATAA**ATGCTTGCTCGTCTTGG
88_1005_down_rev	**ACGCCAAGCTTGCATGCCTGCA**GCTGCTCCAAAGGCAAAC
77_rnpA_fw (qPCR)	GAGTCCGTCAACGAAAGTC
78_rnpA_rev (qPCR)	GTGAGCAGGCCATCAAAG
107_0922_fw (qPCR)	CTGAGCTTGTCGCCATTC
108_0922_rev (qPCR)	GGCAAGGCATTACGGAAG
91_0923_fw (qPCR)	TTCTTTCGGCCTCAGCAG
92_0923_rev (qPCR)	TCGGCCAGTAGTTGACTC
79_1005_fw (qPCR)	GACTGGCACGCTCTTTAC
80_1005_rev (qPCR)	GGCATTCCAAGCCGTATC
105_1006_fw (qPCR)	TCTGCGATCGGCTATCTC
106_1006_rev (qPCR)	TCGCTTGTTGTCCAATCG
109_1004_fw (qPCR)	CGCTTGGGTGTAGTTGAC
110_1004_rev (qPCR)	CCGAGTTCGTGCTGATTC

Chemical synthesis of 5-deoxyribose and 7-deoxysedoheptulose

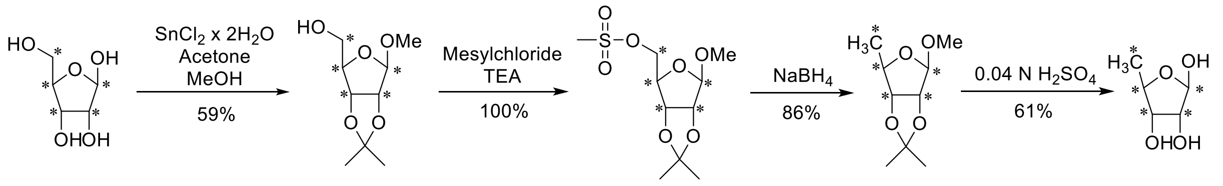

(1) (2) (3) (4) (5)

Figure S6: Four step synthesis of [U-$^{13}C_5$]-5dR modified after(6, 7). (1): $^{13}C_5$-D-Ribose, (2): Methyl-2,3-*O*-isopropylidene-$^{13}C_5$-*β*-D-ribofuranoside, (3): Methyl-2,3-*O*-isopropylidene-5-*O*-mesyl-$^{13}C_5$-*β*-D-ribofuranoide, (4): Methyl-2,3-*O*-isopropylidene-$^{13}C_5$-5-deoxy-*β*-D-ribofuranoide, (5): [U-$^{13}C_5$]-5-Deoxy-D-ribofuranose.

Physiochemical data of intermediates of the chemical synthesis

Abbreviations: TLC: thin layer chromatography; R_f: retention factor; NMR: nuclear magnetic resonance; MHz: megahertz; $CDCl_3$: deuterated chloroform; δ: chemical shift; ppm: part per million; dm: doublet of multiplet; *J*: coupling constants; Hz: hertz; ddm: doublet of doublet of multiplet; d: doublet; s: singlet; m: multiplet; HR-(+)ESI-MS: High resolution-electrospray-mass spectrometry (positive mode); m/z: mass-to-charge ratio; calcd: calculated; ddd: doublet of doublet of doublet; D_2O; deuterium oxide.

Methyl-2,3-*O*-isopropylidene-$^{13}C_5$-β-D-ribofuranoside (2)

TLC: R_f 0.63 (cyclohexane/ethylacetate 1:1)

^{1}H-NMR (400 MHz, $CDCl_3$):
δ (ppm)=4.95 (dm, $J_{1,C\text{-}1}$=174.4 Hz, 1H, 1-H), 4.81 (dm, $J_{3,C\text{-}3}$=160.6 Hz, 1H, 3-H), 4.57 (dm, $J_{2,C\text{-}2}$=158.7 Hz, 1H, 2-H), 4.41 (dm, $J_{4,C\text{-}4}$=155.1 Hz, 1H, 4-H), 3.68 (ddm, $J_{5a,C\text{-}5}$=143.8 Hz, $J_{5a,5b}$=12.6 Hz, 1H, 5a-H), 3.59 (ddm, $J_{5b,C\text{-}5}$=142.0 Hz, $J_{5b,5a}$=12.6 Hz, 1H, 5b-H), 3.42 (d, $J_{\text{-}OCH3,C\text{-}1}$=4.4 Hz, 3H, -O*CH₃*), 1.47 and 1.30 (s, 3H, C*(CH₃)₂*)

^{13}C-NMR (100 MHz, $CDCl_3$):
δ (ppm)=112.2 (*C*(CH₃)₂), 109.8 (d, $J_{C\text{-}1,C\text{-}2}$=48.5 Hz, C-1), 88.4 (dd, $J_{C\text{-}4,C\text{-}3}$=39.8 Hz, $J_{C\text{-}4,C\text{-}5}$=38.9 Hz, C-4), 85.9 (dd, $J_{C\text{-}2,C\text{-}1}$=48.5 Hz, $J_{C\text{-}2,C\text{-}3}$=31.0 Hz, C-2), 81.5 (ddm, $J_{C\text{-}3,C\text{-}4}$=39.8 Hz, $J_{C\text{-}3,C\text{-}2}$=31.0 Hz, C-3), 64.1 (dm, $J_{C\text{-}5,C\text{-}4}$=38.9 Hz, C-5), 55.6 (m, -O*CH₃*), 26.4 and 24.8 (C*(CH₃)₂*)

HR-(+)ESI-MS: *m/z* calcd. for $[M+H]^+$: 210.1238, found: 210.1239; *m/z* calcd. for $[M+Na]^+$: 232.1058, found: 232.1058.

Methyl-2,3-*O*-isopropylidene-5-*O*-mesyl-$^{13}C_5$-β-D-ribofuranoide (3)

TLC: R_f 0.59 (cyclohexane/ethylacetate 1:1)

^{1}H-NMR (400 MHz, $CDCl_3$):
δ (ppm)=4.98 (ddd, $J_{1,C\text{-}1}$=173.9 Hz, $J_{1,C\text{-}2}$=7.5 Hz, $J_{1,2}$=3.0 Hz, 1H, 1-H), 4.69 (dm, $J_{3,C\text{-}3}$=154.5 Hz, 1H, 3-H), 4.60 (ddm, $J_{2,C\text{-}2}$=158.9 Hz, $J_{2,1}$=3.0 Hz, 1H, 2-H), 4.40 (dm, $J_{4,C\text{-}4}$=155.1 Hz, 1H, 4-H), 4.20 (dm, $J_{5a,C\text{-}5}$=152.1 Hz, 1H, 5a-H), 4.18 (dm, $J_{5b,C\text{-}5}$=154.1 Hz, 1H, 5b-H), 3.34 (d, $J_{\text{-}OCH3,C\text{-}1}$=4.5 Hz, 3H, -O*CH₃*), 3.06 (s, 1H, Mesyl-CH₃), 1.47 and 1.30 (s, 3H, C*(CH₃)₂*)

^{13}C-NMR (100 MHz, $CDCl_3$):

δ (ppm)=113.0 ($C(CH_3)_2$), 109.7 (d, $J_{C-1,C-2}$=49.3 Hz, C-1), 85.0 (dd, $J_{C-2,C-1}$=49.3 Hz, $J_{C-2,C-3}$=30.7 Hz, C-2), 83.9 (dd, $J_{C-4,C-5}$=42.5 Hz, $J_{C-4,C-3}$=39.1 Hz, C-4), 81.5 (ddd, $J_{C-3,C-4}$=39.1 Hz, $J_{C-3,C-2}$=30.7 Hz, $J_{C-3,C-5}$=5.5 Hz, C-3), 68.5 (dd, $J_{C-5,C-4}$=42.5 Hz, $J_{C-5,C-3}$=5.5 Hz, C-5), 55.4 (m, -OCH_3), 37.9 (Mesyl-CH_3), 26.5 and 25.0 ($C(CH_3)_2$)

HR-(+)ESI-MS: *m/z* calcd. for $[M+H]^+$: 288.1014, found: 288.1013; *m/z* calcd. for $[M+Na]^+$: 310.0833, found: 310.0831.

Methyl-2,3-*O*-isopropylidene-$^{13}C_5$-5-deoxy-β-D-ribofuranoide (4)

TLC: R_f 0.87 (cyclohexane/ethylacetate 1:1)

^{1}H-NMR (700 MHz, $CDCl_3$):
δ (ppm)=4.92 (ddd, $J_{1,C-1}$=172.3 Hz, $J_{1,C-2}$=7.5 Hz, $J_{1,2}$=2.6 Hz, 1H, 1-H), 4.61 (ddm, $J_{2,C-2}$=161.4 Hz, $J_{2,1}$=2.6 Hz, 1H, 2-H), 4.49 (dm, $J_{3,C-3}$=155.8 Hz, 1H, 3-H), 4.32 (dm, $J_{4,C-4}$=149.6 Hz, 1H, 4-H), 3.31 (d, $J_{-OCH3,C-1}$=4.4 Hz, 3H, -OCH_3), 1.46 and 1.29 (s, 3H, $C(CH_3)_2$), 1.27 (dm, $J_{5,C-5}$=126.3 Hz, 3H, 5-H)

^{13}C-NMR (176 MHz, $CDCl_3$):
δ (ppm)=112.2 ($C(CH_3)_2$), 109.6 (dm, $J_{C-1,C-2}$=48.1 Hz, C-1), 85.9 (dm, $J_{C-2,C-1}$=48.1 Hz, C-2), 85.3 (dm, $J_{C-3,C-4}$=37.7 Hz, C-3), 83.2 (ddm, $J_{C-4,C-3}$=$J_{C-4,C-5}$=37.7 Hz, C-4), 54,5 (m, -OCH_3), 26.6 and 25.1 ($C(CH_3)_2$), 21.0 (dm, $J_{C-5,C-4}$=37.7 Hz, C-5)

HR-(+)ESI-MS: *m/z* calcd. for $[M+H]^+$: 194.1289, found: 194.1294; *m/z* calcd. for $[M+Na]^+$: 216.1109, found: 216.1111.

[U-$^{13}C_5$]-5-Deoxy-D-ribofuranose (5)

TLC: R_f 0.46 (chloroform/methanol 4:1)

^{1}H-NMR (400 MHz, D_2O):
β-furanose: δ (ppm)=5.18 (dm, $J_{1,C-1}$=172.1 Hz, 1H, 1-H), 4.00-3.95 (m, 3H, 2-H, 3-H, 4-H), 1.33 (dm, $J_{3,C-3}$=126.8 Hz, 3H, 5-H)
α-furanose: δ (ppm)= 5.35 (dm, $J_{1,C-1}$=172.6 Hz, 1H, 1-H), 4.14 (dm, $J_{2,C-2}$=151.2 Hz, 1H, 2-H), 4.12 (dm, $J_{4,C-4}$=150.5 Hz, 1H, 4-H), 3.80 (dm, $J_{3,C-3}$=150.2 Hz 1H, 3-H), 1.24 (dm, $J_{3,C-3}$=126.8 Hz, 3H, 5-H)

^{13}C-NMR (100 MHz, D_2O):
β-furanose: δ (ppm)= 100.8 (m, C-1), 78.3 (dm, $J_{C-4,C-5}$=39.5 Hz, C-4), 75.2 (m, C-2 and C-3), 19.1 (d, $J_{C-5,C-4}$=39.5 Hz, C-5)
α-furanose: δ (ppm)= 95.7 (dm, $J_{C-1,C-2}$=42.2 Hz, C-1), 78.1 (dm, $J_{C-4,C-5}$=39.9 Hz, C-4), 74.8 (m, C-3), 70.4 (dm, $J_{C-2,C-1}$=42.2 Hz, C-2), 17.8 (d, $J_{C-5,C-4}$=39.9 Hz, C-5)

HR-(+)ESI-MS: *m/z* calcd. for $[M+Na]^+$: 162.0639, found: 162.0640.

[3,4,5,6,7-$^{13}C_5$]-7-Deoxy-D-*altro*-heptulose

TLC: R_f 0.56 (chloroform/methanol 8:5)

^{1}H-NMR (400 MHz, D_2O):
β-furanose δ (ppm)=4.21 (dm, $J_{4,C-4}$=110.7 Hz, 1H, 4-H), 4.07 (dm, $J_{3,C-3}$=145.4 Hz, 1H, 3-H), 3.94 (m, 1H, H-6), 3.69 (m, 1H, 5-H), 3.63 (dd, $J_{1a,1b}$=11.8 Hz, $J_{1a,C-3}$=4.3 Hz, 1H, 1a-H), 3.54 (d, $J_{1b,1a}$=11.8 Hz, $J_{1b,C-3}$=6.5 Hz, 1H, 1b-H), 1.20 (dm, $J_{7,C-7}$=126.8 Hz, 3H, 7-H)
α-pyranose δ (ppm)=4.07 (m, 1H, 6-H), 4.03 (m, 1H, 4-H), 3.69 (m, 1H, 3-H), 3.66 (m, 1H, 1a-H), 3.57 (m, 1H, 5-H), 3.40 (m, 1H, 1b-H), 1.26 (dm, $J_{7,C-7}$=127.1 Hz, 3H, 7-H)
α-furanose δ (ppm)=4.14 (m, 1H, 4-H), 4.06 (m, 1H, 3-H), 3.99 (m, 1H, 6-H), 3.91 (m, 1H, 5-H), 3.92 (m, 1H, 1a-H), 3.64 (m, 1H, 1b-H), 1.20 (dm, $J_{7,C-7}$=126.8 Hz, 3H, 7-H)

^{13}C-NMR (100 MHz, D_2O):

β-furanose δ (ppm)=101.3 (dm, $J_{C-2,C-3}$=44.4 Hz, C-2), 83.5 (dm, ^{1}J=43.4 Hz, 39.0 Hz, ^{2}J=5.4 Hz, C-5), 75.8 (m, C-3); 74.7 (m, C-4), 67.7 (m, C-6), 62.4 (C-1), 17.0 (d, $J_{C-7,C-6}$=38.4 Hz, C-7)
α-pyranose δ (ppm)=98.0 (m, C-2), 70.8 (dm, ^{1}J=38.9 Hz, C-4), 68.9 (m, C-5), 67.6 (m, C-3), 64.5 (dm, ^{1}J=41.1 Hz, C-6), 63.8 (C-1), 16.9 (m, C-7)
α-furanose δ (ppm)= 104.4 (m, C-2), 84.7 (dm, ^{1}J=40.9 Hz, C-5), 82.0 (dm, $J_{C-3,C-4}$=40.5 Hz, C-3), 75.8 (m, C-4), 66.7 (dm, ^{1}J=38.4 Hz, C-6), 62.9 (m, C-1), 17.0 (d, $J_{C-7,C-6}$=37.4 Hz, C-7)

HR-(+)ESI-MS: *m/z* calcd. for $[M+Na]^+$: 222.0850, found: 222.0852.

Quantification of metabolites in the culture supernatant via GC-MS

To determine the recovery efficiency of the extraction method from the cell pellets (Figure S7 A), as well as from the supernatant (Figure S7 B), we performed spiking experiments with $^{13}C_5$-5dR and $^{13}C_5$-7dSh.

Recovery efficiency in the cell pellet:
For the standard, we added 5dR, $^{13}C_5$-5dR, 7dSh and $^{13}C_5$-7dSh (1000 pmol each) to 700 µL of the extraction solution and proceeded with extraction, derivatization and GC-MS measurement as described in the Materials & Methods section. Additionally, we added $^{13}C_5$-5dR and $^{13}C_5$-7dSh (1000 pmol each) to cell pellets from day 7 (received from 1.5 mL culture) and analogously performed the extraction, derivatization, and GC-MS measurement. The mean of the peak areas of each compound in the standard was set to 100 %. Peak areas of each compound in the cell pellet were normalized to this. Figure S7 A clearly shows that the recovery efficiency of exogenously added $^{13}C_5$-5dR and $^{13}C_5$-7dSh in the cell pellet (grey bars) does not differ from that of the standard (black bars). Besides the measurement of exogenously added ^{13}C-labelled compounds, we simultaneously also measured the unlabeled endogenously produced compounds in these cell pellets. Only 5.34 % of unlabeled 5dR and 1.43 % of unlabeled 7dSh (in relation to the applied 1000 pmol) were found, which corresponds to concentrations measured in Figure S1.

Recovery efficiency in the supernatant:
To determine the recovery efficiency in the supernatant, we added $^{13}C_5$-5dR and $^{13}C_5$-7dSh (1000 pmol each) to 200 µL of culture supernatant, lyophilized the supernatant and proceeded with extraction, derivatization, and GC-MS measurement as described in the Materials & Methods section. The standard, containing 5dR, $^{13}C_5$-5dR, 7dSh and $^{13}C_5$-7dSh (1000 pmol each) was also lyophilized and then treated as the supernatant sample. The mean of the peak area of each compound in the standard was set to 100 %. Peak areas of each compound in the cell pellet were normalized to this. Figure S7 B clearly shows that the recovery efficiency of exogenously added $^{13}C_5$-5dR and $^{13}C_5$-7dSh in the supernatant (grey bars) does not differ from that of the standard (black bars). Besides the measurement of exogenously added ^{13}C-labelled compounds, we simultaneously also measured the unlabeled endogenously produced compounds in the supernatant of day 14. 218 % of unlabeled 5dR and 6 % of unlabeled 7dSh were found (in relation to the applied 1000 pmol), which corresponds to concentrations measured at this time point.

These experiments show that the extraction and derivatization method described in the Material & Methods section is applicable to culture supernatant as well as cell pellets.

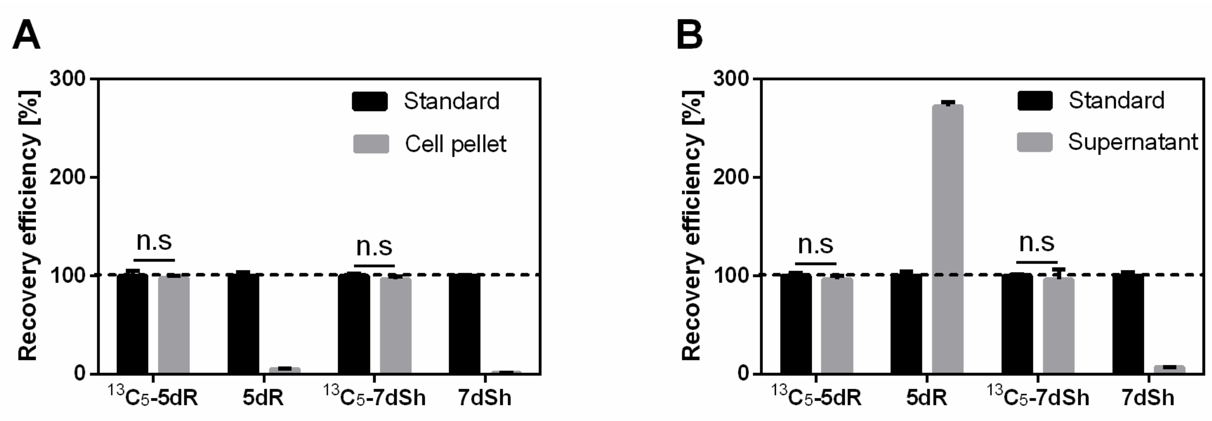

Figure S7: Recovery efficiency of exogenously added $^{13}C_5$-5dR and $^{13}C_5$-7dSh and the amount of unlabeled, endogenously formed 5dR and 7dSh (in relation to the standard containing 1000 pmol of the respective substance) during extraction and derivatization of the cell pellet (A) and the supernatant (B). Mean of peak areas of each compound in the standard was set to 100 %. Values shown in the graph represent mean and standard deviation of three replicates. Significant differences between the recovery efficiency of exogenously added $^{13}C_5$-5dR and $^{13}C_5$-7dSh in the standard and in the cell pellet (A) or in the supernatant (B) were analyzed by using an unpaired t-test (* p-value < 0.05; ** p-value < 0.01; *** p-value < 0.001; n.s.: not significant).

References

1. Ma L, Bartholome A, Tong MH, Qin Z, Yu Y, Shepherd T, Kyeremeh K, Deng H, O'Hagan D. 2015. Identification of a fluorometabolite from *Streptomyces* sp. MA37: (2*R*3*S*4*S*)-5-fluoro-2,3,4-trihydroxypentanoic acid. Chem. Sci. 6:1414–1419. doi:10.1039/C4SC03540B.
2. Sekowska A, Danchin A. 2002. The methionine salvage pathway in *Bacillus subtilis*. BMC Microbiol 2:8. doi:10.1186/1471-2180-2-8.
3. Sekowska A, Denervaud V, Ashida H, Michoud K, Haas D, Yokota A, Danchin A. 2004. Bacterial variations on the methionine salvage pathway. BMC Microbiol 4:9. doi:10.1186/1471-2180-4-9.
4. Kanehisa M, Goto S. 2000. KEGG: Kyoto Encyclopedia of Genes and Genomes. Nucleic Acids Res 28:27–30. doi:10.1093/nar/28.1.27.
5. Beaudoin GAW, Li Q, Folz J, Fiehn O, Goodsell JL, Angerhofer A, Bruner SD, Hanson AD. 2018. Salvage of the 5-deoxyribose byproduct of radical SAM enzymes. Nat Commun 9:3105. doi:10.1038/s41467-018-05589-4.
6. Sairam P, Puranik R, Sreenivasa Rao B, Veerabhadra Swamy P, Chandra S. 2003. Synthesis of 1,2,3-tri-*O*-acetyl-5-deoxy-D-ribofuranose from D-ribose. Carbohydr. Res. 338:303–306. doi:10.1016/S0008-6215(02)00464-0.
7. Zhang JT, Chen SP, Feng JM, Liu DW, Tang LJ, Wang XJ, Huang SP. 2013. Synthetic Study of 1, 2, 3-Tri-*O*-Acetyl-5-Deoxy-D-Ribofuranose. Adv Mat Res 781-784:1184–1186. doi:10.4028/www.scientific.net/AMR.781-784.1184.
